# Taming hemoglobin chemistry—a new hemoglobin-based oxygen carrier engineered with both decreased rates of nitric oxide scavenging and lipid oxidation

**DOI:** 10.1038/s12276-024-01323-x

**Published:** 2024-10-01

**Authors:** Chris E. Cooper, Michelle Simons, Alex Dyson, Nélida Leiva Eriksson, Gary G. A. Silkstone, Natalie Syrett, Victoria Allen-Baume, Leif Bülow, Luca Ronda, Andrea Mozzarelli, Mervyn Singer, Brandon J. Reeder

**Affiliations:** 1 School of Life Sciences, University of Essex, Wivenhoe Park, Colchester, Essex UK; 2 Centre for Pharmaceutical Medicine Research, Institute of Pharmaceutical Science, King’s College London, London, UK; 3 Pure and Applied Biochemistry, Department of Chemistry, Lund University, Lund, Sweden; 4 Department of Medicine and Surgery, University of Parma, Parma, Italy; 5 Institute of Biophysics, National Research Council (CNR), Pisa, Italy; 6 Department of Food and Drug, University of Parma, Parma, Italy; 7 Bloomsbury Institute for Intensive Care Medicine, Division of Medicine, University College London, London, UK; 8 Present Address: Biotechnology, Department of Chemistry, Lund University, Lund, Sweden

**Keywords:** Biochemistry, Medical research, Biologics

## Abstract

The clinical utility of hemoglobin-based oxygen carriers (HBOC) is limited by adverse heme oxidative chemistry. A variety of tyrosine residues were inserted on the surface of the γ subunit of recombinant fetal hemoglobin to create novel electron transport pathways. This enhanced the ability of the physiological antioxidant ascorbate to reduce ferryl heme and decrease lipid peroxidation. The γL96Y mutation presented the best profile of oxidative protection unaccompanied by loss of protein stability and function. N-terminal deletions were constructed to facilitate the production of recombinant hemoglobin by fermentation and phenylalanine insertions in the heme pocket to decrease the rate of NO dioxygenation. The resultant mutant (αV1del. αL29F, γG1del. γV67F, γL96Y) significantly decreased NO scavenging and lipid peroxidation in vitro. Unlike native hemoglobin or a recombinant control (αV1del, γG1del), this mutation showed no increase in blood pressure immediately following infusion in a rat model of reperfusion injury, suggesting that it was also able to prevent NO scavenging in vivo. Infusion of the mutant also resulted in no meaningful adverse physiological effects apart from diuresis, and no increase in oxidative stress, as measured by urinary isoprostane levels. Following PEGylation via the Euro-PEG-Hb method to increase vascular retention, this novel protein construct was compared with saline in a severe rat reperfusion injury model (45% blood volume removal for 90 minutes followed by reinfusion to twice the volume of shed blood). Blood pressure and survival were followed for 4 h post-reperfusion. While there was no difference in blood pressure, the PEGylated Hb mutant significantly increased survival.

## Tyrosine and Phenylalanine Mutations Create Safer Hemoglobin for Oxygen Delivery

Hemoglobin-based oxygen carriers are modified hemoglobin molecules that can be infused as blood substitutes to replace red blood cell transfusions or as oxygen therapeutics to deliver oxygen to damaged tissues not readily accessible by red cells. However, their clinical use has been limited by adverse side effects caused by free radical production and nitric oxide scavenging by extracellular hemoglobin. The researchers used genetic engineering to insert tyrosine residues into fetal human hemoglobin to decrease radical production and phenylalanine residues to decrease nitric oxide scavenging. The resulting novel hemoglobin was tested in rat models to observe the effects on blood pressure and survival rates. The research offers hope for improved treatment for patients in critical need of blood transfusions or with an otherwise compromised oxygen delivery system, such as in sickle cell disease, stroke or sepsis.

This summary was written by the author.

## Introduction

Artificial hemoglobin (Hb)-based oxygen carriers (HBOCs) have long had potential as sterile, stable, long-lasting products that are able to transport oxygen to tissues^1,2^. Initially, HBOCs were designed to be transfused at high doses in place of packed red blood cells to restore impaired oxygen transport under acute conditions when blood was not readily available (a “blood substitute”). More recent interest has focused on the use of HBOCs transfused at lower doses to deliver oxygen more efficiently to sites where red blood cells cannot reach^3^. This could be due to the abnormalities of red cell deformability observed in sickle cell crisis^4^ or the disordered microvascular circulation observed in trauma, sepsis and other acute inflammatory states^5^. HBOC may also be able to facilitate transport to oxygen-deprived tissues in cases of regional tissue hypoxia, such as in subarachnoid hemorrhage or stroke^6^. When used in this way as an “oxygen therapeutic,” HBOC products not only deliver bulk oxygen but also facilitate oxygen transfer from red cells to hypoxic tissue^7^.

However, whether used as a blood substitute or an oxygen therapeutic, HBOCs need to be able to deliver their oxygen safely without unwanted chemical reactions that have the potential to damage tissue. Concern over tissue toxicity, rather than a lack of efficacy in oxygen transport, has been the reason HBOCs have not been approved more generally for clinical use^8^. This toxicity is generally driven by the uncontrolled chemistry of hemoglobin outside the protective environment of red blood cells.

There are three main reasons why the hemoglobin molecule is potentially toxic when present in plasma^9^. First, ferrous (Fe^2+^) oxyhemoglobin (OxyHb) reacts rapidly with the intercellular messenger nitric oxide, causing vasoconstriction and an increase in blood pressure^10^. Second, OxyHb autoxidizes to ferric (Fe^3+^) methemoglobin (metHb), which can undergo Fe^3+^/Fe^4+^ (ferric/ferryl) redox cycling driven by hydrogen and/or lipid peroxides. This can cause extensive oxidative damage to lipids, DNA, and proteins^11^. Finally, there is a slow rate of heme loss, especially from ferric metHb^12,13^; free heme acts as a damage-associated molecular pattern (DAMP) protein^14^ with the potential to perturb the immune system, e.g., via complement activation^15^. Creating a viable HBOC therefore means “taming” this chemistry, in effect making the protein more resistant than the native Hb to a less favorable extracellular environment.

We have recently inserted tyrosine residues on the surface of adult Hb to add new through-protein electron transfer pathways (TPETPs), facilitating the ability of plasma antioxidants to prevent oxidative stress due to ferryl/ferric redox cycling. Crucially, these mutants do not perturb Hb functional activity^16^. Other mutations increase the ability of metHb to be reduced to the functional OxyHb form by plasma antioxidants such as ascorbate^17^.

**Fig. 1:**
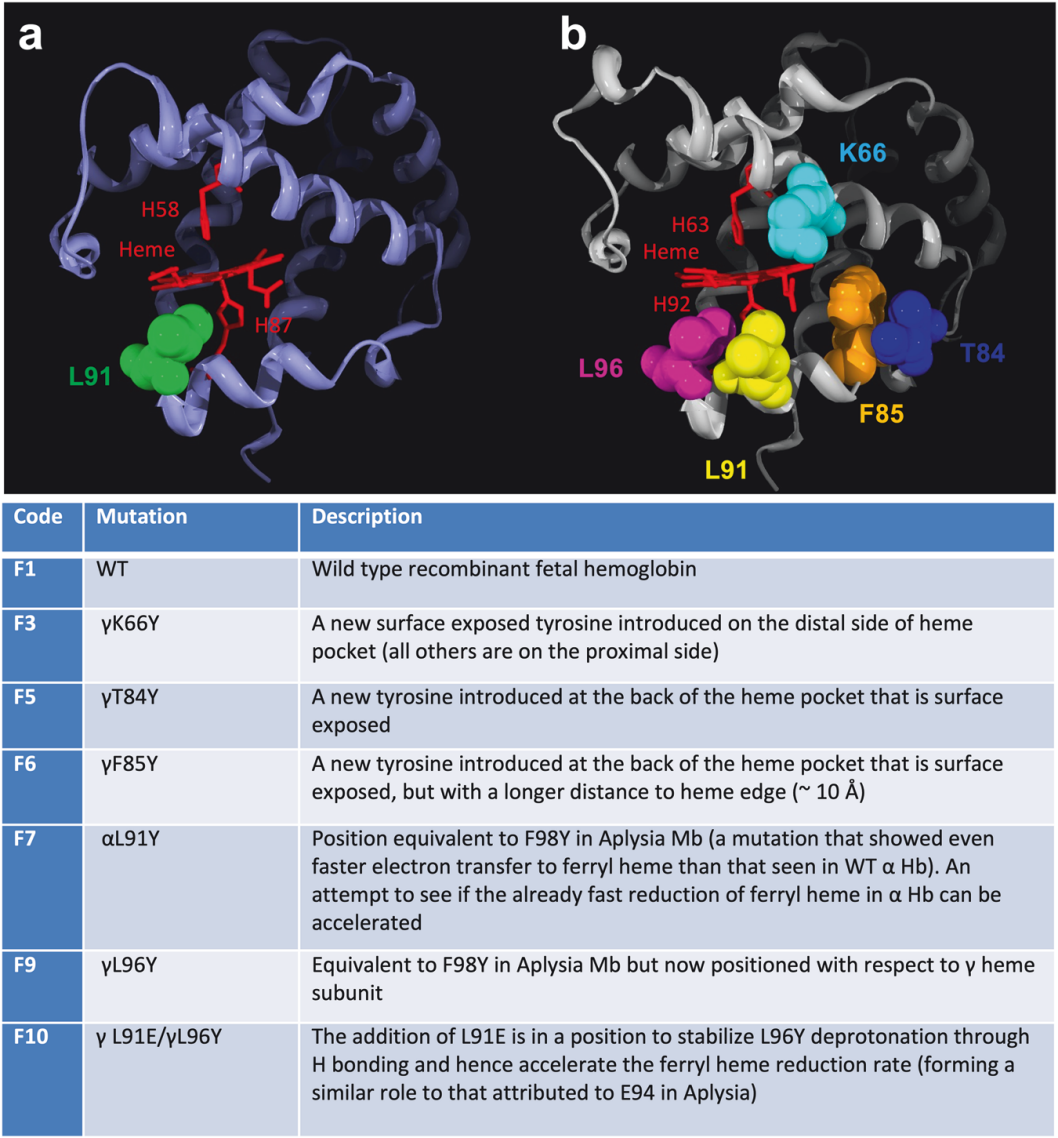
Sites chosen for the addition of through-protein electron transfer pathways. The three-dimensional α- and γ-chain structures of HbA, illustrating the position of the novel mutations introduced. The structure file 4MQJ from the Protein Data Bank was used to generate the figure. **a** α chain L91 mutation point shown in green. **b** γ chain mutation points: K66 (cyan) is on the distal side of the heme, and T84 (blue), F85 (orange), L91 (yellow), and L96 (pink) are on the proximal side of the heme. Heme is shown in a red stick representation with the proximal and distal histidines. F1-F10 are code names later used in the text for the mutants described.

In this study, the aim was to introduce similar surface-facing tyrosine mutations in the more stable fetal form of Hb. The selection process for creating the optimal HBOC was to first examine the effect of adding new TPETPs to fetal hemoglobin, primarily in the γ subunit, followed by additional mutations in the lead mutant to increase stability and decrease NO scavenging^10^. To enhance the reduction of the high oxidation states (ferryl and ferric) of Hb by plasma antioxidants, mutations in TPETP require proximity to the heme iron while being surface exposed. Here, specific residues that fit these characteristics were targeted for the introduction of an electron transfer pathway and individually mutated to redox-active tyrosine residues. The rationale is described in Fig. 1 and is similar to that used previously to enhance TPETP in adult human hemoglobin^16,17^ and *Aplysia fasciata*^18^. The α chain of Hb already possesses a tyrosine (Tyr 42), which enhances ferryl reduction^19^. A tyrosine mutation at αL91 was introduced to determine whether additional TPETP enhanced the ferryl reduction already present. The γ chain for HbF (or the β chain for HbA) has no endogenous tyrosine residue to enable electron transfer. Sites on the proximal and distal sides of the heme pocket were therefore identified as potential new pathways in the γ subunit.

In the next stage, mutations were added to increase the production of Hb by deleting the N-terminal methionine residues (αV1del. γG1del.). Owing to the deficiency of methionine cleavage in *E. coli*, these deletions are necessary to prevent extension of the N-terminus and hence facilitate the large-scale production of recombinant Hb by fermentation^20^. Mutations were then added to decrease NO scavenging by decreasing the volume of the distal heme pocket via the addition of aromatic amino acids in place of leucine and valine^21^. Tryptophan is the most effective amino acid for decreasing NO scavenging and lowering blood pressure (BP)^10^. However, we chose to insert phenylalanine instead in an attempt to prevent the enhanced heme loss observed in Hb mutants incorporating tryptophan^22^.

Short-term effects on blood pressure (BP) and oxidative stress were tested in a rat model of moderate reperfusion injury. To measure longer-term effects on BP and survival time, a more severe model was used, and mutants were PEGylated at multiple (6-8) surface amino residues using the Euro-PEG-Hb method to enable greater plasma retention^23^.

In vitro and in vivo studies suggest that this novel recombinant product has the potential to be the first of a new class of HBOCs with significantly fewer toxic side effects.

## Materials and methods

### Protein expression and purification

Native (nonrecombinant) adult human Hb was purified from human volunteers as described previously^24^. For details of the recombinant protein expression, purification and PEGylation, see the supplementary information (Protein Methods).

### Optical spectroscopy

Unless stated otherwise, all optical spectra were taken using a Cary 5000 spectrophotometer (Agilent). To produce the various forms of Hb, carbonmonoxyHb was first oxidized to the metHb (ferric Fe^3+^) form by the addition of excess potassium ferricyanide while under constant illumination with a strong but low-temperature light source that acted to photolyze CO from ferrous iron. The ferricyanide was then removed by gel filtration through a PD-10 column (Cytiva). The deoxyHb form was made by the addition of a slight excess of sodium dithionite to the metHb form. This was then applied to a PD-10 column to generate OxyHb. All Hb concentrations used in this study are reported in heme molar equivalents.

### p50 measurements

For proteins F1-F10, p50 measurements were carried out in 100 mM HEPES buffer, pH 7.0, 100 mM sodium chloride, 1.2 mM sodium phosphate, and 1 mM EDTA. The final protein concentration was 100 µM. Before dilution, the samples were centrifuged to remove precipitates. Oxygen equilibrium curves were measured at 25 °C, as previously reported^25^. For each sample, the absorption spectrum (equilibrated in air) was collected immediately after thawing. The Hayashi enzymatic reducing system was added to the solution before titration to reduce metHb and to limit its formation during titration. The samples were subsequently deoxygenated via a helium flow and then equilibrated with different oxygen partial pressures.

The oxygen affinities of proteins F25 and F45 were measured in 100 mM sodium phosphate buffer, pH 7.0, at 20 °C. Approximately 100 µM OxyHb was deoxygenated under vacuum (purged with argon) and then equilibrated with increasing volumes of oxygen. The fractional saturation (Y) was calculated. Hill plots were generated by plotting log(pO_2_) vs. log(Y/(1-Y)), and the p50 and Hill coefficient were calculated from the x-intercept and gradient, respectively.

### Autoxidation

The autoxidation of 10 µM OxyHb in 70 mM sodium phosphate buffer, pH 7.2, at 37 °C was monitored optically. The OxyHb concentrations were calculated using the extinction coefficient 125,000 M^−1^ cm^−1^ (415 nm)^26^. Time courses (405–500 nm) were fitted to single exponential functions, minimizing the least squares difference, by using the Microsoft Excel Solver function.

### Heme release

The metHb forms of the proteins (4 µM) were incubated with hemopexin (4.5 µM) obtained from human plasma (Sigma) in 10 mM sodium phosphate buffer, pH 7.2, at 37 °C. The concentration of the metHb proteins was calculated optically using the extinction coefficient of 179,000 M^−1^ cm^-1^ (405 nm) for the H_2_O-bound high-spin form at pH ~7. The time courses (401–418 nm) were fitted to single exponential functions, minimizing the least squares, by using the Microsoft Excel Solver function.

### Ferric reduction

MetHb (20 μM) in 70 mM sodium phosphate buffer, pH 7.2, was reacted at a 1:1 volume ratio with varying concentrations of sodium ascorbate at 25 °C. The time courses (577–630 nm) were fitted to a single exponential function, and rates were plotted against the ascorbate concentration. The second-order rate constant was determined by fitting to a straight line.

### Ferryl formation and reduction

metHb (10 µM) in 70 mM sodium phosphate buffer, pH 7.2, was reacted with a 3x excess of H_2_O_2_ at 25 °C to produce the ferrylHb (Fe^4+^) forms. The reaction of the metHb proteins with H_2_O_2_ was monitored optically to ensure the formation of the ferryl forms, and to determine the rate of ferryl formation. The time courses (425-406 nm) were fitted to a single exponential function using Kaleidagraph ^TM^ version 4.5.2.

Catalase was added to remove excess H_2_O_2_ and the mixture was incubated for ~10 seconds to fully react. Sodium ascorbate was then added at a 1:1 volume ratio so that the final concentration of Hb was 5 µM. The reactions were then followed to completion using an Agilent 8453 diode array spectrophotometer. The time courses (425–406 nm) were fitted to double exponential functions, minimizing the least squares, by using Microsoft Excel Solver. For each time course, the two calculated rate constants were assigned to the reactions of the alpha and gamma subunits and the data were plotted as a function of the reductant concentration for each protein. This ascorbate concentration-dependent kinetic profile was fitted to a rectangular hyperbola plus a straight line with an offset to the measured autoreduction rate (Kaleidagraph ^TM^ version 4.5.2).

### Reactions with HPODE

The lipid hydroperoxide 13S-hydroperoxy-9Z,11E-octadecadienoic acid (HPODE) was produced as previously described^27^. MetHb (10 µM) was reacted with 100 µM, 50 µM, and 25 µM HPODE in 70 mM sodium phosphate buffer, pH 7.2. Kinetics were measured in a rapid mixing stopped-flow instrument, and the reactions were monitored using a diode array at 24 °C. Time courses (405–700 nm) were fitted to single exponentials, minimizing the least squares, by using Microsoft Excel Solver.

### Lipid oxidation

Alpha-phosphatidylcholine (5 mg/mL) derived from soybean (Type II-S, Sigma) in 10 mM sodium phosphate buffer, pH 7.2, was sonicated in a water bath for approximately 5 minutes until no particulates could be observed. To produce unilamellar liposomes 100 nm in diameter, this suspension was then passed through a liposome extruder (Northern Lipids) containing a membrane with a size cutoff of 0.1 µm (Whatman) 10 times. The liposomes were stored at 4 °C and used within 4 h.

MetHb (2 µM) was incubated with 200 µg/ml liposomes for 10 h at 25 °C. Sodium ascorbate was added at a final concentration of 50 µM as indicated. Lipid oxidation was monitored by following the production of conjugated dienes by measuring the absorbance at 234 nm using a Tecan Infinite M200Pro plate reader. The lag time before the onset of lipid oxidation was then determined.

### NO scavenging

An NO solution was prepared by dissolving the NO donor ProliNONOate (Cayman Chemical Company) in 25 mM NaOH. The concentration of ProliNONOate was determined using an extinction coefficient of 8500 M^−1^ cm^−1^ at 250 nm (when added to the buffer at neutral pH, 1.8 molecules of NO are released per ProliNONOate molecule). The ProliNONOate was then diluted into thoroughly degassed 70 mM sodium phosphate buffer, pH 7.2. OxyHb (10 μM) was reacted at a ratio of 1:1 with various concentrations of ProliNONOate in a rapid mixing stopped-flow spectrophotometer (Applied Photophysics). Owing to the speed of the reaction, the temperature was set to 15 °C. The reactions were monitored at 422 nm and fitted to a single exponential. The data were plotted as a function of NO concentration for each protein, and the second-order rate constants were determined by fitting to a straight line.

### Animal studies

For general animal husbandry, instrumentation and analytical methods, see Supplementary Fig. 1 and the supplementary file (Animal Methods). Two different protocols were used (Supplementary Fig. 2).

### Study 1 and Study 2 – administration of unPEGylated Hb following hemorrhage

Following a 1-h stabilization period post-surgery, one-third of the estimated blood volume (based on 70 mL/kg) was removed from the carotid arterial line over 10 min into heparinized 5 ml syringes. The animals were further monitored for 20 min prior to resuscitation. At resuscitation, for Study 1, the animals received either Hb (2.5 mL/300 g body weight; 2.48 mM heme) or an equivalent volume of Ringer’s lactate, which was administered over 5 min. At resuscitation, for Study 2, two different types of purified recombinant fetal Hb were compared (F25 and F45). Eight animals per group were studied. Blood pressure (BP) was measured at baseline and at the end of ischemia and immediately following the end of Hb or vehicle administration. Studies 1 and 2 were identical, except that Study 1 was completed prior to Study 2 to ensure that the model was of sufficient severity to show meaningful changes in blood pressure and oxidative stress without compromising animal survival.

### Study 3 – efficacy of PEGylated F45 (pF45) following severe hemorrhage

Following a 1-h stabilization period post-surgery, 45% of the estimated blood volume (based on 70 mL/kg) was removed from the carotid arterial line over 15 min into heparinized 5 ml syringes. The animals were further monitored for 75 min (total ischemia time = 90 min) prior to randomization.

During the ischemia phase, if the BP fell below 40 Torr between 30 and 90 min from the onset of hemorrhage, a fluid bolus (1 mL of Ringer’s lactate solution) was administered. This enables a greater proportion of animals to remain alive until resuscitation. The animals then received either PEGylated F45 (2.5 mL/300 g body weight over 5 min; 3 mM heme; 8.33 mL/kg) or an equivalent volume of Ringer’s lactate. Sixteen animals per group were studied.

Additional fluids were given over the next 15 min such that all animals received twice the volume of shed blood by the end of resuscitation, i.e., 90% estimated blood volume (63 mL/kg) minus 8.33 mL/kg (p45/Ringer’s lactate) = 54.67 mL/kg. In this survival study, the animals were monitored for up to 4 h after the onset of resuscitation. Mortality within this timeframe is defined by cardiac arrest.

BP was measured at baseline, at the end of ischemia, after Hb/vehicle administration, after Ringer’s lactate fluid infusion, and then hourly after the onset of resuscitation. Core temperature measurements, echocardiography measurements, and arterial blood gas measurements were performed at baseline, at the end of ischemia, 1 h after the onset of resuscitation and at the end of the experiment (4 h). Urine output was determined between 0–1 and 1–4 h post-resuscitation. A blood sample (1 ml; replaced with 2 ml of Ringer’s lactate) was taken at 1 h post-resuscitation, before the onset of significant mortality. All of the plasma and urine samples were stored in liquid nitrogen during experimentation and in a −80 °C freezer thereafter prior to batch analysis.

### Statistics

The statistical tests used are described above or in the relevant place in the Table and Figure legends.

## Results

### In vitro biochemical studies

**Fig. 2:**
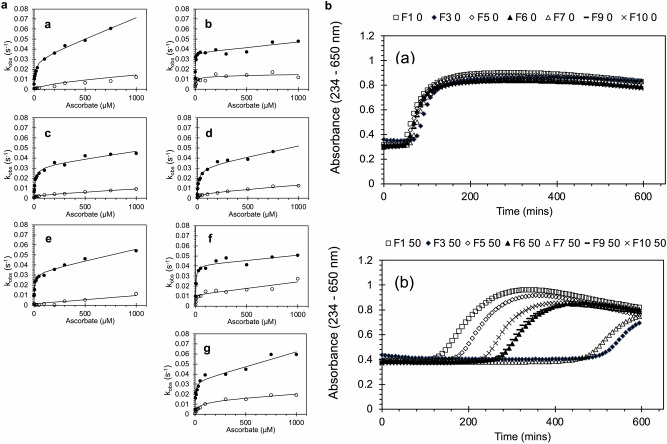
Biochemical properties of fetal Hb designed to decrease oxidative stress. **a** Effect of mutations on the rate constant for ferryl reduction: α chain (closed circles) and γ chain (open circles). **a** WT (F1), (**b**) F3 γK66Y, (**c**) F5 γT84Y, (**d**) F6 γF85Y, (**e**) F7 αL91Y, (**f**) F9 γL96Y, and (**g**) F10 γL91E/γL96Y. **b** Effect of mutations on lipid oxidation: metHb (2 µM) was reacted with phosphatidylcholine liposomes in 70 mM sodium phosphate buffer, pH 7.2, ± 50 µM ascorbate, and the formation of conjugated dienes was measured at an absorbance of 234 nm to calculate the lag time before oxidation (*n* = 3). **a** Average conjugated diene formation with time in the absence of reductant; (**b**) average conjugated diene formation with time in the presence of 50 µM ascorbate.

Each TPETP mutation was evaluated on the basis of the ability of ascorbate to reduce the peroxide-induced cytotoxic ferryl species to ferric (Fig. 2a and Table 1). In the wild-type (WT) recombinant rHbF (F1) protein, both the α and γ chains showed an autoreduction rate of ~ 0.005–0.01 s^−1^, where the ferryl heme iron acquires an electron from its surroundings, manifested as a non-zero y-axis intercept with no ascorbate added. However, the two chains differ with respect to the saturable ascorbate concentration dependence of ferryl reduction. In the absence of mutations, faster kinetics are observed in the α chain due to the presence of the endogenous redox-active tyrosine (αTyr42). This exhibits a kinetic profile as a function of ascorbate concentration that can be modeled by a hyperbolic function representing a high-affinity TPETP pathway for heme reduction and a lower-affinity direct reaction modeled as a second-order electron transfer between the reductant and the heme iron. The latter direct (non-ascorbate saturable, non-tyrosine mediated) pathway for heme reduction is relevant to the overall kinetics only at higher, non-physiological levels of ascorbate. Although generally higher in the α subunit, it is of the same order of magnitude in the γ subunit and varies only slightly between mutants.

**Table 1:** Ferryl reduction by ascorbate.

	α subunit				γ subunit			
	*V*_max_ (s^−1^)	*K*_m_ (µM)	Linear (M^−1^ s^−1^)	*V*_max_/*K*_m_ (μM^−1^ s^−1^)×10^−3^	*V*_max_ (s^−1^)	*K*_m_ (µM)	Linear (M^−1^ s^−1^)	*V*_max_/*K*_m_ (μM^−1^ s^−1^)×10^−3^
F1	0.0244 ± 0.0019	15.7 ± 3.53	41.9 ± 3.6	1.55 ± 0.37	0.0056 ± 0.0056	227.8 ± 300.3	8.99 ± 4.70	0.025 ± 0.041
F3	0.0250 ± 0.0018	2.71 ± 1.11	11.0 ± 3.4	9.22 ± 3.84	0.0102 ± 0.0021	13.2 ± 9.38	1.93 ± 3.20	0.77 ± 0.57
F5	0.0259 ± 0.0021	17.4 ± 4.55	14.9 ± 3.1	1.49 ± 0.41	0.0022 ± 0.00043	46.0 ± 21.6	6.13 ± 0.52	0.048 ± 0.024
F6	0.0270 ± 0.0026	15.2 ± 4.57	21.6 ± 5.1	1.78 ± 0.56	0.0037 ± 0.00074	40.0 ± 19.9	8.80 ± 0.92	0.093 ± 0.05
F7	0.0234 ± 0.0017	11.7 ± 3.03	23.2 ± 2.8	2.00 ± 0.54	Not measurable	Not measurable	10.5 ± 0.95	Not measurable
F9	0.0308 ± 0.0023	5.35 ± 2.03	10.1 ± 4.0	5.76 ± 2.23	0.0097 ± 0.0015	4.6 ± 3.80	12.8 ± 2.7	2.11 ± 1.77
F10	0.0280 ± 0.0027	5.12 ± 2.13	29.3 ± 4.4	5.47 ± 2.34	0.0120 ± 0.0026	37.1 ± 18.9	7.52 ± 3.18	0.32 ± 0.18

The rate constants of ferryl reduction by the α and γ subunits were fitted to a rectangular hyperbola plus a straight line, offsetting the measured autoreduction rates to obtain the *V*_max_ and *K*_m_ plus a linear rate constant. Notably, the high fit values and errors indicate that this approach is unsuitable for the γ subunit in the absence of a tyrosine insert (F1 and F7; in the latter case, the errors are an order of magnitude larger than the values and are not recorded in the table). Errors in *V*_max_ and *K*_m_ are SEMs of the nonlinear curve fit. The SEM of the ratio *V*_max_/*K*_m_ was approximated from the respective SEM, assuming that the variables were independent and using a first-order Taylor expansion. Var(x/y) =x^2^/y^2^ × (Var(x)/x^2^ + Var(y)/y^2^).

In the α chain, the TPETP kinetics of all of the mutants reached a maximum of ~ 0.026 s^−1^, with K_m_ values between 2.7 and 15.7 µM (Table 1). Compared with F1, the addition of a tyrosine residue in this chain (F7) did not alter the *V*_max_ or the *K*_m_, suggesting that a single tyrosine (in this case, Tyr42) is all that is necessary to enable optimal electron transfer from ascorbate to the ferryl heme in the α subunit. However, some mutations in the γ subunit do perturb the rate of electron transfer via αTyr42, as evidenced by slightly higher values of *V*_max_/*K*_m_ in F3, F9, and F10.

In contrast, and like the homologous adult β chain, the fetal γ chain shows no high-affinity saturable phase in the absence of TPETP mutations (F1 and F7), with any *K*_m_ either having an error of measurement similar to (F1) or significantly greater than (F7) the *K*_m_ itself. However, all of the newly introduced tyrosine residues in the γ chain facilitate a significantly high-affinity saturable rate, although still with a lower *V*_max_ and *V*_max_/*K*_m_ compared to the α chain rates. The highest catalytic efficiency (*V*_max_/*K*_m_) was observed for F9 and F3.

Figure 2b shows that there was no difference in the rate of lipid oxidation in the absence of ascorbate in the fetal mutants. However, in the presence of physiological levels of ascorbate (50 µM), the addition of novel surface tyrosine residues significantly delayed the onset of lipid oxidation, as measured by the formation of conjugated dienes (see also Table 2). In the presence of ascorbate, liposome oxidation was significantly inhibited by all tyrosine mutations, with F3 and F7 showing the greatest effects. The subunit into which the tyrosine is inserted seems less relevant for the ability of ascorbate to prevent lipid oxidation than for ferryl reduction, as F7 (αL91Y) had longer lag times than many of the γ subunit insertions.

**Table 2:** Positive and negative attributes of all mutations.

	Oxygen affinity p50 (torr)	Hill coefficient (h)	Autoxidation (min^−1^)	Heme release (min^−1^)	Ferric reduction (M^−1 ^min^−1^)	Ferryl formation (s^−1^)	HPODE reactivity (s^−1^)	Liposome oxidation lag (min)
**F1**	6.4 ± 0.1	2.53 ± 0.16	0.010 ± 0.003	0.054 ± 0.014	1.53 ± 0.16	12.76 × 10^−3^ ± 1.27 × 10^−4^	5.60 × 10^−2^ ± 7.55 × 10^−4^	123.38 ± 2.25
**F3**	5.6 ± 0.2	1.95 ± 0.13	0.007 ± 0.0006	0.141 ± 0.025**	1.10 ± 0.0032	19.38 × 10^−3^ ± 5.78 × 10^−4^	9.74 × 10^−2^ ± 8.19 × 10^−4^	513.40 ± 3.63***
**F5**	8.0 ± 0.3	2.04 ± 0.17	0.023 ± 0.001*	0.059 ± 0.004	1.49 ± 0.074	8.82 × 10^−3^ ± 9.08 × 10^−5^	4.48 × 10^−2^ ± 5.78 × 10^−4^	173.20 ± 0.52***
**F6**	5.3 ± 0.3	1.81 ± 0.22	0.011 ± 0.001	0.080 ± 0.011	2.04 ± 0.076	9.99 × 10^−3^ ± 1.07 × 10^−4^	5.44 × 10^−2^ ± 6.59 × 10^−4^	267.27 ± 0.3***
**F7**	19.8 ± 0.4	1.84 ± 0.11	0.016 ± 0.003	0.089 ± 0.008*	5.93 ± 0.51	21.02 × 10^−3^ ± 1.36 × 10^−3^	8.59 × 10^−2^ ± 1.38 × 10^−3^	463.96 ± 2.55***
**F9**	8.3 ± 0.3	1.66 ± 0.1	0.011 ± 0.002	0.074 ± 0.011	1.79 ± 0.064	14.75 × 10^−3^ ± 3.84 × 10^−4^	8.36 × 10^−2^ ± 1.19 × 10^−3^	263.15 ± 1.18***
**F10**	8.4 ± 0.4	1.28 ± 0.09	0.008 ± 0.0004	0.097 ± 0.008**	0.97 ± 0.12	10.02 × 10^−3^ ± 1.89 × 10^−4^	9.70 × 10^−2^ ± 7.18 × 10^−4^	229.00 ± 1.90***

For details of the assays, see the “Methods” section. For p50, ferric reduction, ferryl formation, and HPODE reactivity, the mean ± SEM was directly calculated from nonlinear fitting of the experimental data at different reactant concentrations. For autoxidation, heme release, and liposome oxidation lag times, the data are presented as the means ± SDs for studies performed in triplicate. In these latter cases, significance was compared to the F1 control (only) via an unpaired Student’s *t* test *p < 0.05; **p < 0.01; ***p < 0.001.

Table 2 shows the effects of the tyrosine mutations on the functional features of Hb. All of the mutations showed reversible, cooperative oxygen binding (oxygen affinity, p50), although with a slightly decreased cooperativity compared with that of the WT (decreased h compared with F1). The only change in affinity of a relevant magnitude was that of F7 (αL91Y), which bound oxygen almost three times as weakly as the WT. F7 was the only mutant to show a meaningful increase in the rate of ferric reduction by ascorbate (>tripling), although the rate was still significantly slower than that observed in adult Hb.

Table 2 also shows the effects on the kinetics of the properties of Hb deleterious to HBOCs (autoxidation, heme release, ferryl formation, and lipid oxidation via HPODE or liposomes). Most noticeable here is the increased autoxidation rate ( > doubled) of F5 and the increased (tripled) rate of heme loss in F3. Smaller but possibly still meaningful increases in heme loss were observed for F7 and F10. F7 also had the highest rate of ferryl formation. In agreement with the effects on liposome oxidation in the absence of ascorbate (Fig. 2b), the oxidation rates of the lipid HPODE were rather similar in all of the mutants.

In choosing a mutation to take forward to the next stage of development, we compared the activities across the range of measured attributes, focusing first and foremost to prevent negative attributes that might be exacerbated as further mutations were added. F3, F7, and F10 were therefore ruled out due to increased heme release; F5, for increased autoxidation; and F7, for decreased oxygen affinity. Among the remaining two mutants, F6 and F9 had broadly similar functional attributes (Table 2), but F9 was significantly better at introducing a high-affinity ferryl reduction in the γ subunit (Fig. 2a and Table 1), the rationale for introducing new tyrosine-based electron transfer sites in the first place. Therefore, F9 (γL96Y) was chosen as the backbone for further mutations in the putative fetal HBOC.

**Fig. 3:**
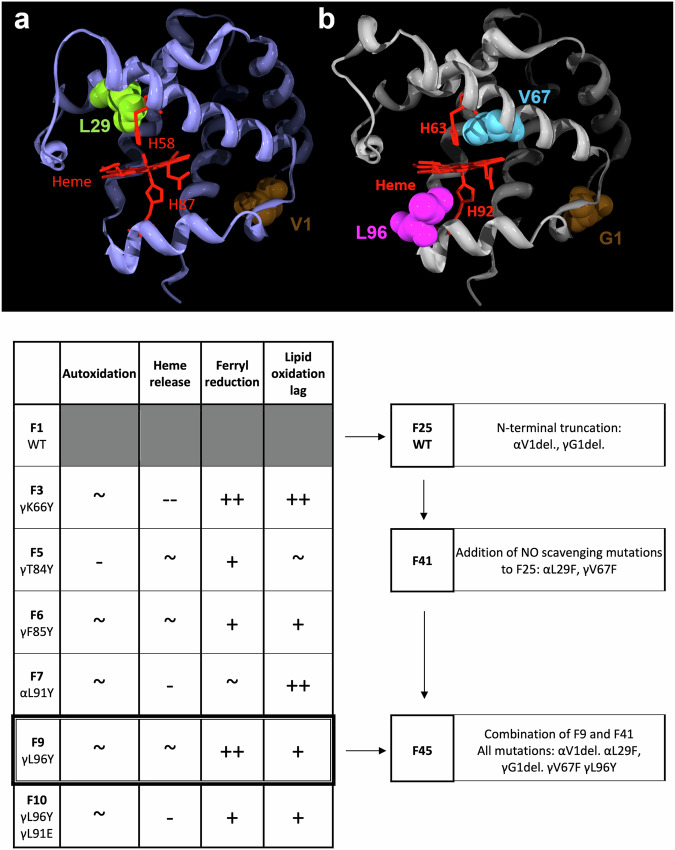
Rationale for the selection of mutations in the final HBOC product. All mutant proteins from Fig. 1 were compared to the WT (F1) protein and assigned symbols according to their potential benefits as a feature of an HBOC. A positive symbol indicates an increase in a desirable property/a decrease in an undesirable property, and a negative symbol indicates a decrease in a desirable property/increase in an undesirable property. ~ comparable to WT, (**-**)**-** (much) worse than WT, (+)+ (much) better than WT. This suggested F9 as the best option going forward. F9 was combined with N-terminal truncation (F25) and mutations designed to alter the NO scavenging properties of the protein (F41) to make F45 (α chain (**a**), γ chain (**b**), image at top).

The overall strategy to produce the “lead” combination of mutations (F45) is illustrated in Fig. 3. N-terminal deletions were made in the α and γ subunits in the recombinant WT (F1) to generate a new F25 control; then, phenylalanine was introduced into α and γ to decrease NO scavenging (F41), followed by tyrosine to introduce a new TPETP in the γ subunit (F45).

**Fig. 4:**
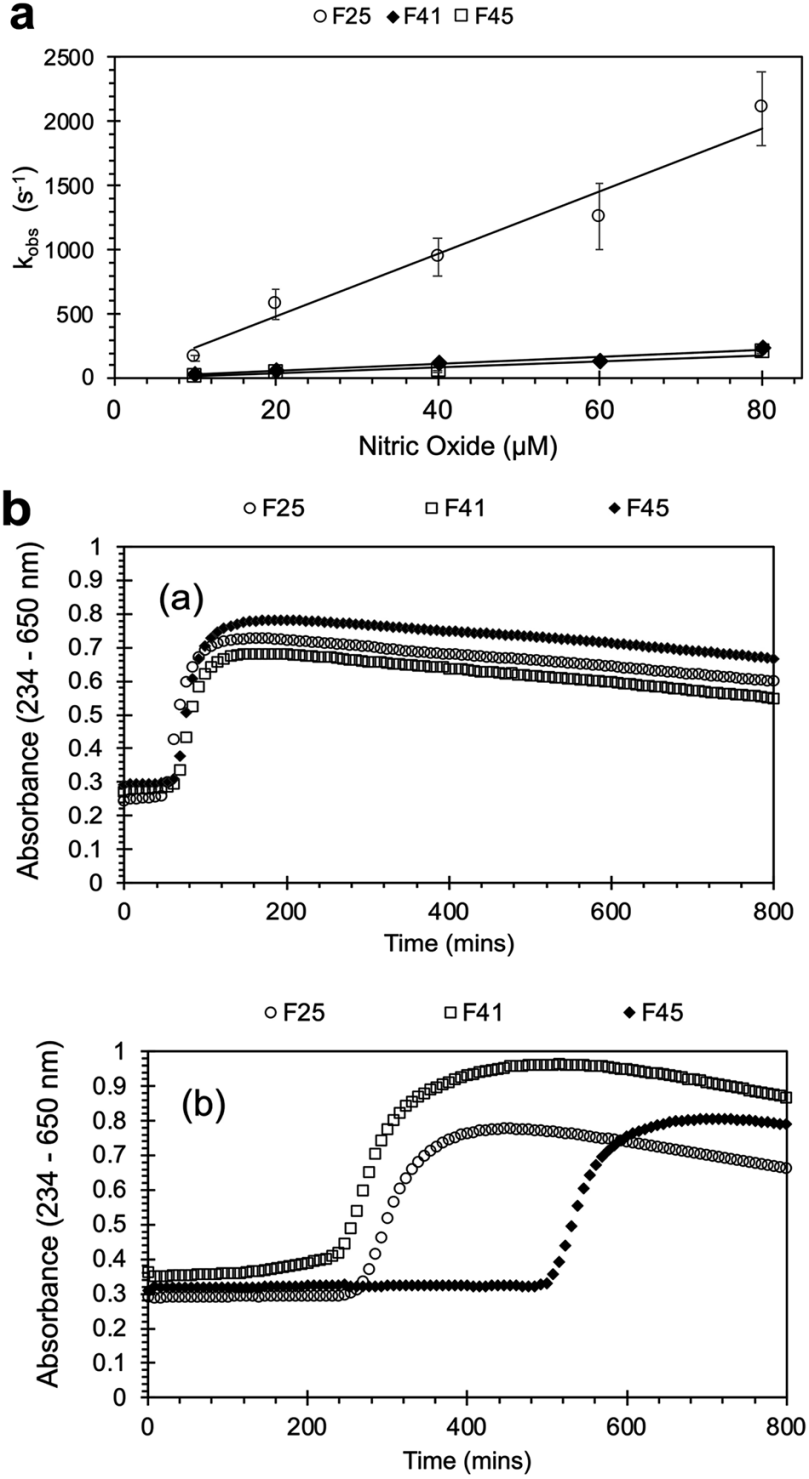
Biochemical properties of fetal Hb designed to decrease NO scavenging. **a** Rate of NO dioxygenation: OxyHb (5 μM) was reacted with NO in 70 mM sodium phosphate buffer, pH 7.2, at 15 °C. The rate constants were determined by fitting to a single exponential function and were plotted against the NO concentration (mean ± SD, *n* = 4). The second-order rate constants were then determined by fitting to a straight line: F25 open circles; F41 filled diamonds; and F45 open squares. **b** Rate of lipid oxidation: metHb (2 µM) was reacted with phosphatidylcholine liposomes in 70 mM sodium phosphate buffer, pH 7.2, ± 50 µM ascorbate, and the formation of conjugated dienes was measured at an absorbance of 234 nm to calculate the lag time before oxidation (*n* = 3). **a** Average conjugated diene formation with time in the absence of reductant; (**b**) average conjugated diene formation with time in the presence of 50 µM ascorbate. F25 open circles; F41 open square; F45 filled diamond.

Figure 4a shows that the insertion of Phe residues into the heme pocket in F41 significantly decreased the rate of NO dioxygenation by OxyHb. The additional insertion of a Tyr mutation to introduce a TPETP (F45) resulted in a similar decrease in the rate of NO scavenging. However, the presence of the additional tyrosine resulted in decreased lipid oxidation in the presence of ascorbate, with an increased lag phase of liposome oxidation in F45 compared to both F41 and F25 (Fig. 4b).

Table 3 shows the biochemical activities relevant to a putative HBOC for F45 compared with F25 (as a control) and F41. Compared with the WT protein, the addition of mutations to decrease NO scavenging successfully decreased the rate of NO dioxygenation 10-fold, with no meaningful increase in the rates of autoxidation or heme loss. F45 reversibly bound to oxygen in a cooperative manner, albeit with a slightly higher affinity than both “control” Hbs (F1 and F25). While the addition of a surface tyrosine in F45 significantly decreased the rate of lipid oxidation compared with that in F41 and F25, it was not possible to measure whether it had a similar positive effect on the ascorbate reduction of ferryl; the phenylalanine mutations in the heme pocket in F41 and F45 appeared to prevent the formation of a stable ferryl intermediate following the addition of hydrogen peroxide, making it impossible to test for the presence of a TPETP in the way shown in Fig. 2a. There was a significant increase in heme loss in F41 and F45 compared with F25, although the rate was still lower than that in the recombinant WT F1, as shown in Table 2.

**Table 3:** Comparison of the functional properties of F25, F41, and F45.

		F25	F45	F41
p50	p50 (torr)	7.91	4.34	ND
	Hill coefficient (h)	1.9	1.5	ND
Autoxidation (min^−1^)		0.0022 ± 0.0005	0.0058 ± 0.0028***	0.0151 ± 0.0012***
Heme release (min^−1^)		0.0300 ± 0.0092	0.0495 ± 0.0093*	0.0487 ± 0.0075*
Ferric reduction (M^−1 ^min^−1^)		2.10 ± 0.095	10.32 ± 0.045	9.14 ± 0.82
Liposome oxidation lag (min)		269.1 ± 15.4	509.9 ± 11.7***	243.4 ± 8.9*
NO dioxygenation (μM^−1^s^−1^)		25.68 ± 1.11	2.69 ± 0.47***	2.89 ± 0.17***

For details of the assays, see the methods section. ND, not determined. For ferric reduction, the mean ± SEM was directly calculated from nonlinear fitting of the experimental data to different concentrations of oxygen and ascorbate. For autoxidation, heme release, liposome oxidation lag time, and NO dioxygenation, the data are presented as the mean ± SD for studies performed in triplicate. In these latter cases, significance was compared to the F25 control (only) via an unpaired Student’s *t* test *p < 0.05; ***p < 0.001.

### Moderate reperfusion injury rat model: Study 1 (native human Hb vs. vehicle) and Study 2 (F25 vs. F45)

**Fig. 5:**
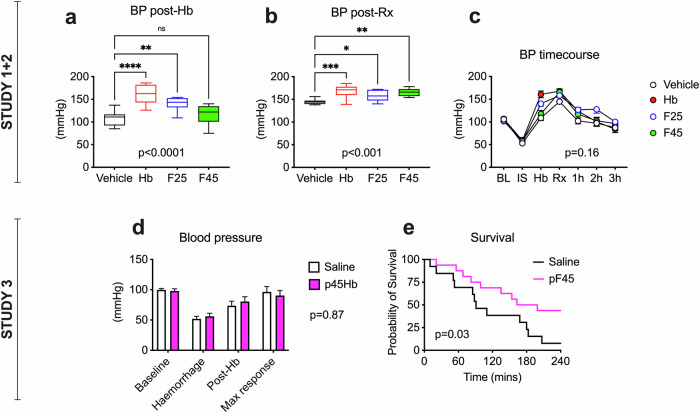
Effects of Hb and PEGylated Hb on BP and survival in a rat model of reperfusion injury. Studies 1 and 2 compared vehicle (PBS) with unPEGylated OxyHb (native, F25 and F45) in a moderate rat reperfusion injury model. Changes in BP immediately following Hb and then subsequent blood reinfusion (Rx) are shown in (**a**) and (**b**), respectively. The complete BP time course for Studies 1 and 2 is shown in (**c**). Study 3 compared PEGylated F45 (pF45) to a vehicle (saline) control in a severe rat reperfusion injury model. BP and survival are shown in (**d**) and (**e**), respectively. One-way ANOVA followed by Dunnett’s multiple comparisons test was used in (**a**) and (**b**). Two-way ANOVA was performed in (**c**) and (**d**), followed by Bonferroni’s multiple comparisons test in (**c**). A log-rank test was performed in (**e**). The actual *P* values are the result of the overall test. For post hoc analyses in (**a**) and (**b**), *p < 0.05, **p < 0.01, ***p < 0.001. BL baseline, BP blood pressure, Hb hemoglobin, IS ischemia, Rx all treatment (Hb + blood).

All of the animals (*n* = 32) survived until the end of the experiment. With the exception of diuresis, no significant adverse events were observed with native human Hb, F25 or F45 (Supplementary Figs. 3–9). The BP measurements are shown in Fig. 5a–c. The removal of one-third of the blood volume caused a 50% decrease in BP. The subsequent addition of Hb with unmodified NO scavenging properties (native Hb, F25) resulted in a significant increase in BP. This was not observed with F45. Post-reperfusion, BP was significantly greater in all Hb-treated animals than in the vehicle-treated animals. The BP of all of the animals then decreased slowly over time post-resuscitation. At the end of the experiment, the BPs for all of the treatment groups were comparable to the baseline values.

Respiration rates and core temperature were similar across all time points and treatment groups, although urine output was significantly elevated in native Hb-treated animals and those treated with F25 but not with F45 (Supplementary Fig. 3). Blood loss caused an expected decrease in stroke volume (Supplementary Fig. 4). Heart rates also decreased, as did cardiac output. Cardiac contractility was lower following hemorrhage, as evidenced by a decrease in aortic peak systolic blood flow velocity. Administration of Ringer’s lactate + autologous blood reversed all of the deficits associated with blood loss by 1-h post-resuscitation. There were no statistically significant differences in cardiovascular performance between F25 and F45 (Supplementary Fig. 4).

Blood loss induced several other expected physiological changes (Supplementary Fig. 5). Arterial hemoglobin levels fell with increased ventilation (increases in PaO_2_) and hemoglobin oxygen saturation (SaO_2_) and a decrease in arterial PaCO_2_. Catecholamine-induced (due to blood loss) glycogenolysis and insulin resistance caused a spike in blood glucose levels (Supplementary Fig. 6); the reduction in oxygen delivery was indicated by an increase in blood lactate levels and metabolic acidosis revealed by reductions in arterial base excess. The arterial pH remained largely unchanged due to compensation by the respiratory component of acid‒base balance (increased ventilation). Resuscitation by all treatments reversed these derangements. There were no significant differences between the treatment groups in Study 1. Study 2 revealed differences in arterial pH that were significantly greater in F25-treated animals than in control animals but were not clinically relevant. All other electrolytes remained unchanged, within the normal range in both studies, and similar across treatment groups (Supplementary Fig. 7).

Pharmacokinetic analyses revealed plasma half-lives of 39.6, 83.2, and 115.5 min for native Hb, F25, and F45, respectively (Supplementary Fig. 8). The maximum plasma concentration differed among the treatments in the following order: F45 > F25 > (native) Hb. This value was significantly greater for F45 than for the other two treatment groups. The proportion of metHb also differed between the treatments. No native metHb was present in the plasma samples from Study 1 (native Hb); however, over the time course of the entire experiment, 16.2% and 36.1% of total Hb was present as metHb for F25 and F45, respectively. Disappearance of Hb from the bloodstream appears dependent on Hb oxidation status, with the oxidized form demonstrating a longer half-life.

The unmodified Hb molecules used in Studies 1 and 2 were rapidly renally excreted (Supplementary Fig. 9). The majority of Hb and F25 were excreted in the first hour after the onset of resuscitation, which coincided with a high urine output in this time window. Total renal excretion (over the time course) was between 25% and 30% for Hb- and F25-treated animals. In contrast, only 1.3% of F45 was renally excreted, contributing to the higher plasma concentrations and slower half-life observed with this hemoglobin form. The oxidation status of renally excreted Hb also differed between treatments. The levels of native Hb and F25 were similar, with the majority of Hb excreted as OxyHb. In contrast, in F45-treated animals, a greater proportion of met-Hb was excreted, albeit in far lower quantities than in the other two treatments. Notably, (on average), we could only observe up to 30% of the total Hb being renally excreted. This is notwithstanding plasma concentrations being far lower in the latter half of the experiment; in Study 1, the plasma Hb concentration decreased 97% by 3 h.

When time-dependent concentrations were assessed, urinary levels of oxidative stress, as assayed by isoprostane concentrations, were not different between the treatment groups in Study 1 (Supplementary Fig. 10). However, total isoprostane levels (corrected for urine volume) were significantly elevated in native Hb-treated animals in the first hour after the onset of resuscitation. Although total isoprostanes in F45 were lower than those in the F25 control, this difference did not reach significance, possibly because of the large variability in the urine output of the recombinant proteins.

### Severe reperfusion injury survival model (Study 3, PEGylated F45 vs. vehicle)

Figure 5d, e illustrates the changes in BP over time and survival. Compared with the saline control, the infusion of PEGylated F45 (pF45) did not significantly affect BP. However, survival at 4 h significantly increased following the addition of pF45.

## Discussion

The results of this study demonstrate that it is possible to design a recombinant fetal Hb with in vitro antioxidant properties and decreased NO scavenging without perturbing normal physiological function; initial in vivo animal studies suggested that these mutations could form the basis for future HBOCs that could be used clinically.

All of the introduced tyrosine residues on the surface of the γ subunit created a new TPETP, accelerating ascorbate reduction of ferryl heme. The observed increases were all significant but of differing magnitudes. As the redox-active Tyr residues are all closer than 17 Å from the heme, the distance itself is unlikely to be a factor in the differing rates^28^, suggesting instead small changes in reorganization energy or redox potential. It has been proposed that electron transfer occurs (via a Marcus mechanism^18^) only within a small subpopulation of the protonated oxoferryl and a deprotonated tyrosine. Hence, small perturbations of the pK for tyrosine deprotonation could have significant effects on TPETP.

The γL91E/γL96Y double mutation (F10), which is designed to facilitate tyrosine deprotonation and increase TPETP, had no advantageous effects over the single γL96Y (F7) mutation. It is difficult to generalize properties from the predicted structure, as the magnitude of increase in the γ mutations was different from that seen in the homologous mutations in the β subunit in the adult protein^17^. For these reasons, a phenomenological approach was used in taking the design forward.

All of the introduced tyrosine residues decreased the rate of metHb-catalyzed liposome oxidation by extending the lag phase typically observed in this assay^16,17,29^. This is a complex reaction, but it is generally assumed that ferric/ferryl redox chemistry drives oxidation. As with the adult mutant βF41Y, the increase in the size of the lag phase was observed only in the presence of ascorbate^16^. On their own, tyrosine mutations are ineffective. This finding suggests that TPETP itself cannot block the redox cycling that drives liposome oxidation and only facilitates the ability of external reductants to function effectively. Clearly, more than one ferric/ferryl redox cycle is required to initiate the autocatalytic phase of liposome oxidation; the presence of surface tyrosines able to facilitate electron transfer between the reductant and the ferryl heme short circuits this process. However, the lack of a strong correlation between the rate of ferryl reduction in the γ subunit mutants and the length of the lag phase suggests that the situation is more complex. As does the situation of the introduced tyrosine in the α subunit (F7), which does not increase ferryl reduction yet still delays liposome oxidation in the presence of ascorbate. It is possible that this tyrosine can transfer electrons to the ferryl heme more effectively when Hb is bound to liposomes, possibly due to a change in redox potential, as can happen when heme proteins bind to lipid membranes^30^. Alternatively, the F7 tyrosine residue can enable a pathway from the unpaired electron on a lipid peroxide radical to ascorbate without engaging the heme, as previously suggested to explain similar anomalous findings in *Aplysia* myoglobin^18^.

Consistent with data from adult Hb^10,31^, there is a decrease in the rate of NO dioxygenation when bulky aromatic residues are introduced into the heme pockets of the fetal α and γ subunits. Crucially, the decrease is identical in the presence (F45) or absence (F41) of a surface tyrosine TPETP mutation. F41 and F45 introduce phenylalanine residues in both the α and γ heme pockets (αL29F and γV67F, respectively). This decreases the rate of NO deoxygenation ten-fold. This is meaningful, although it is not as large as the almost forty-fold decrease observed when leucine and, especially, tryptophan residues are introduced. However, that larger increase came at the expense of significantly enhanced autoxidation and heme release when measured at 37 °C^22^. This likely contributed to the fact that the tryptophan heme pocket mutations present in the rHb3011 HBOC produced by Baxter International, Inc., did not proceed beyond phase 1 clinical trials^32^. Unlike rHb3011, F45 does not exhibit excess autoxidation or heme loss at 37 °C.

Our previous results suggest that there is little difference in vivo in the use of fetal or adult Hb as a starting material for HBOCs^33^. However, fetal Hb (HbF) is intrinsically more stable^34^ and ultimately is likely to be easier to mass produce^35^; it also seems less likely to cause DNA damage^36^. One area where HbF is unfavorable is that the reduction of metHb to OxyHb by plasma reductants such as ascorbate is an order of magnitude slower^33^. However, this low rate is increased 4–5-fold in F41 and F45; it is possible that a change in redox potential^37^ has enabled autoxidized metHb to be reduced more readily to functional OxyHb in these mutants.

HbF is competent to deliver oxygen in adult humans: HbF comprises an average of 25% of total Hb in asymptomatic adult humans with sickle cell disease in Eastern Arabia^38^; pharmacological induction of the HbF gene is therapeutic in both sickle cell disease and β-thalassemia^39^. Although HbF has a different oxygen affinity and response to external effectors than the adult protein does, its p50 is likely to be just as easy to manipulate as other oxygen-transporting globins, where mutations can tune the p50 by almost 100-fold if desired^40^. In this respect, it is important that TPETP and NO dioxygenase mutants have relatively little effect on the p50, enabling further fine-tuning as necessary for different clinical conditions.

Compared with Ringer’s lactate administration, the infusion of free native Hb rapidly increased the mean arterial BP. A similar effect was observed when control recombinant Hb (F25) was infused. However, no increase was observed in the case of F45, with phenylalanine mutations in the heme pocket. The effects of infusing Hb with different NO scavenging abilities have been extensively studied by Olson’s group^10,21^. Our data are consistent with their findings that decreasing NO attenuates the immediate increase in BP following Hb infusion in a rat model.

Extracellular Hb most likely increases BP via extravascular translocation of Hb into interstitial spaces, including vascular smooth muscle cells^41^. This promotes vascular NO resistance. One mechanism of preventing this is by making the cell-free Hb large enough to prevent this extravasation, as favored by many HBOCs^42^. A key advantage of using recombinant tools to decrease the intrinsic NO scavenging rate in an HBOC is that this effect will persist wherever the HBOC migrates post-administration. In the absence of size-selective compartmentalization, unPEGylated F45 (Study 2) presumably still extravasates, similar to native Hb. However, once it enters interstitial spaces, it is less effective at removing NO, thus preserving the signaling pathway.

While being a good indicator of whether an HBOC scavenges NO, preventing an immediate BP increase by preserving NO signaling does not guarantee the safety of an HBOC^43,44^. In some clinical situations, such an increase might actually be desirable. Indeed, HBOCs have been explicitly designed with the intention of increasing BP in conditions such as septic shock^45^. It is still unclear how HBOCs ultimately damage human organs^9,42,46^. However, long-term perturbation of NO signaling is clearly one potential mechanism^8^.

To explore these longer-term effects, we compared PEGylated F45 (pF45) with Ringer’s lactate in a rat reperfusion injury model. pF45 had no effect on BP, as expected from the short-term study using unPEGylated Hb. More importantly, enhancing the vascular retention of HBOCs via PEGylation increased the survival rate over a 4-h period. Previous HBOCs have had similar positive effects in short-term survival models^47^ but have failed in phase II/III clinical trials^46^.

Whether the strategy of modifying the Hb chemistry at the protein source, rather than by chemical modifications of native Hb, fares any better in further preclinical and clinical studies remains to be determined. However, recombinant and chemical modifications do not need to be exclusive. Recombinant Hb is a template onto which chemical modifications can be added. Engineering a protein with decreased intrinsic NO scavenging when extravasated does not preclude the additional security of polymerizing the molecule into a giant Hb to decrease extravasation in the first place^48^. The creation of an intrinsic antioxidant, TPETP, does not preclude the subsequent addition of antioxidant enzymes^49^ or nanozymes^50^ or co-administration with antioxidant molecules such as ascorbate^51^ or N-acetyl cysteine^52^. Taming Hb chemistry with such an enhanced library of chemical and genetic modifications provides renewed optimism for the role of HBOCs as oxygen therapeutics.

## Supplementary information

Animal Methods

Protein Methods

Supplementary Figures (animal studies)
