# Supplementary material for: Taming hemoglobin chemistry—a new hemoglobin-based oxygen carrier engineered with both decreased rates of nitric oxide scavenging and lipid oxidation
Source: Exp Mol Med. 2024 Oct 1;56(10):2260–70. doi: 10.1038/s12276-024-01323-x (PMC11542024; doi:10.1038/s12276-024-01323-x)
Supplement: Supplementary file 1 — Animal Methods [file 12276_2024_1323_MOESM1_ESM.pdf]

General methodology (Supplementary Fig. 1)

Animals and husbandry

All experiments were performed at the Bloomsbury Institute of Intensive Care Medicine, Division of Medicine, University College London, London. Male Wistar rats (approximately 300 g body weight) were used in all studies. Ethical approval for this work was provided by our local ethics committee (University College London) and UK Home Office guidelines under the Animals (Scientific Procedures) Act 1986; PPL 70/8290: Organ dysfunction following acute illness. Animals were purchased from Charles River Laboratories (Margate, UK) and certified healthy and pathogen-free. Prior to experimentation, animals were housed in cages of four individuals on a 12-h light/dark cycle, with food and water ad libitum prior to experimentation. Standard cages and bedding were used. Additional tissue paper was provided for comfort, and cardboard tubes for cage enrichment.

Instrumentation

Spontaneously breathing animals were anaesthetized by 5% isoflurane (Abbott, Maidenhead, UK) in room air (reduced to 2% post-induction) and placed on a heated mat to maintain rectal temperature at 37 °C. Inhaled isoflurane was used as it allows better cardiorespiratory stability over other agents in spontaneously-breathing animals. Figure S1 shows an illustration of the surgical instrumentation. The left common carotid artery and right internal jugular vein were cannulated using 0.96 mm outside diameter PVC tubing catheter (Scientific Commodities Inc, Lake Havasu City, AZ, USA). The arterial line was connected to a pressure transducer (Powerlab; AD

Instruments, Chalgrove, UK) for continuous monitoring of mean arterial pressure. The venous line was used for subsequent administration of fluids, autologous blood and hemoglobin, as required. A tracheostomy was sited using 2.08 mm external diameter polythene tubing (Portex Ltd, Hythe, UK) to secure and suction the airway. This was connected to a T-piece to maintain anaesthesia. A keyhole laparotomy was performed; the bladder was subsequently cannulated for drainage and quantification of urine output. At the end of surgery, maintenance anaesthesia was provided by 1.5% isoflurane in room air.

Echocardiography

Transthoracic echocardiography, to determine myocardial function, was performed using a 14 MHz probe scanning at 0–2 cm depth (Vivid[i], GE Healthcare, Bedford, UK). Aortic blood flow velocities were determined in the aortic arch using pulsed-wave Doppler. Stroke volume was determined as the product of the velocity–time integral (VTI) and vessel cross-sectional area. Heart rate and systolic peak blood flow velocity (the latter a marker of left ventricular contractility and shortened to ‘peak velocity’) were determined by measuring the time between, and maximum blood flow velocity, respectively, of six consecutive cardiac cycles. Cardiac output was calculated as the product of stroke volume and heart rate. Respiration rates were determined by time-motion imaging of the diaphragm.

Arterial blood gas analysis

Whole blood (100 µL) was collected from the carotid arterial line into heparinised capillary tubes (Brand GMBH, Wertheim, Germany) and inserted into a blood-gas

analyser (ABL 90 FLEX, Radiometer, Crawley, UK). This allowed measurement of partial pressures of oxygen (PaO_2) and carbon dioxide ($PaCO_2$), pH, hemoglobin (and percentage oxygen bound), glucose, lactate and electrolytes (K^+, Na^+, Ca^{2+}, Cl^-). It additionally calculates arterial base excess; this was used as a marker of metabolic acidosis, defined as the quantity of acid or base required to titrate a litre of blood back to physiological pH.

Blood and organ collection

Whole blood was removed from the arterial line and transferred to Eppendorf tubes containing heparin to prevent clotting (20 IU/mL). This was centrifuged at 14000 rpm for 1-min and the plasma fraction subsequently stored at -80 ^{o}C in standard 2 mL cryogenic vials until batch analysis. Whole blood removed for blood depletion was withdrawn in an identical fashion into 5 mL syringes. In studies where autologous blood was reinfused, these syringes are kept at physiological temperature (37 ^{o}C) on a heated mat until administration. At the end of each experiment of all three studies, 2 ml blood was removed and processed for future study.

Pharmacokinetic measurements in plasma and urine

Pharmacokinetic measurements were enabled by the absorbance of free hemoglobin in plasma and urine samples. For plasma samples, where no methemoglobin (metHb) was present, absorbance wavelength was set to the lambda max of 577 nm, with oxyhemoglobin (OxyHb) concentrations derived from standard curves of purified human blood. In plasma and urine samples containing a mixture of Oxy- and metHb, the latter was assessed at 635 nm and derived against standard curves of blood oxidized by K-ferricyanide (10 mM, 2-mins incubation at room temperature). The test

samples were then additionally oxidized by K-ferricyanide, as above, and total hemoglobin (as metHb) was assessed at 635 nm. The oxyHb component in test samples was then derived by subtracting metHb (prior to K-ferricyanide treatment) from total Hb (post-oxidation). Background absorbance artefacts of all samples were removed by subtracting absorbance at 700 nm. All samples were assessed using a microplate reader and BioTek (Gen5) software (Synergy 2, North Star Scientific, Sandy, UK). With knowledge of the number of moles administered, and calculation of the number of moles that appear in urine during the course of each experiment, a mass-balance approach was used to calculate percentage renal excretion.

Molecular markers of renal injury

The extent of potential oxidative damage to the kidney was measured using assays that detect F2 isoprostanes. These stable prostaglandin-like compounds are formed in vivo via peroxidation of arachidonic acid and can be detected using ELISAs. We measured F2 isoprostanes in urine using an OxiSelect™ 8-iso-Prostaglandin F2a ELISA Kit as a marker of renal injury (Cambridge Bioscience, Cambridge, UK). Samples were performed in duplicate according to the manufacturers' instructions.

Data and statistics

Data are presented as mean ± SEM or median, IQR and range. Data sets were compared using an unpaired, non-parametric t-test (Mann-Whitney) or a 2-way repeated measures ANOVA followed by Bonferroni's multiple comparison test. Probability values <0.05 were considered statistically significant.
