# Supplementary material for: Taming hemoglobin chemistry—a new hemoglobin-based oxygen carrier engineered with both decreased rates of nitric oxide scavenging and lipid oxidation
Source: Exp Mol Med. 2024 Oct 1;56(10):2260–70. doi: 10.1038/s12276-024-01323-x (PMC11542024; doi:10.1038/s12276-024-01323-x)
Supplement: Supplementary file 2 — Protein Methods [file 12276_2024_1323_MOESM2_ESM.pdf]

Protein Methods

Fetal wild type and mutant hemoglobins (F1 – F10) were expressed and purified as described previously [1]. Truncated fetal wild type and mutant hemoglobins (F25 – F45) were expressed as above and purified as follows. Cell pellets were resuspended in 20 mM Tris pH 8.5 and lysed using an Avestin C3 Emulsiflex homogenizer. The cell lysate was cleared by centrifugation at 38000 g for 30 min at 4 °C and the pH was adjusted to 8.5 by the addition of small quantities of potassium hydroxide. The centrifugation step was then repeated, and the lysate filtered through a 0.45 µm syringe filter before being loaded on to a 5 mL HiTrap IMAC column (Cytiva) charged with zinc acetate. The column was washed with 20 mM Tris pH 8.5, 500 mM NaCl, then 200 mM Tris pH 8.5, then 20 mM Tris pH 8.5 and finally Hb was eluted with a 10 column volume gradient of Tris pH 8.5, 30 mM EDTA. Selected fractions were then pooled and buffer exchanged using Amicon ultra-15 30 kDa MWCO spin concentrators (Millipore). Once the volume was < 1 mL it was loaded on to a 5 ml Q-HP column (Cytiva) pre-equilibrated with 20 mM Tris pH 8.5. Protein was eluted with a 10 column volume gradient of 50 mM sodium phosphate, pH 7.2, 100 mM sodium chloride. Finally, Hb was concentrated using an Amicon Ultra 30 kDa MWCO and then buffer exchanged into 70 mM sodium phosphate, pH 7.2. All buffers used during the purification procedure were bubbled with CO and the lysate and proteins were bubbled with CO at every stage. Four to fifteen percent of gradient TGX stain free precast gels (Bio-Rad) were used to assay purity following each column stage. The concentration of the ferrous CO (carbonmonoxyHb) bound form of Hb was calculated using an extinction coefficient at 419 nm of 191,000 $M^{-1}cm^{-1}$ and stored in liquid nitrogen.

The mutant Hb used in the *in vivo* animal studies, were produced in a bioreactor (5L, 10L, or 200L depending on the demand). For this, a pre-inoculum was grown overnight at 30 °C in LB media containing 100 µg/mL ampicillin. This pre-inoculum was used to inoculate the fermentation starter which was incubated at 37 °C with shaking until OD_{600} was ~1.0 and then used to inoculate the bioreactors according to Looker *et al.* [2]. The volumes of the pre-inoculum and the starter were 1% and 10% of the volume of the medium in the bioreactors, respectively.

Glucose was used as the carbon source and the expression of hemoglobin was induced by decreasing the temperature to 22 °C and by adding IPTG (Sigma-Aldrich) and aminolevulinic acid (Molekula) in a single shot. The pH was maintained using 5 M NH_4OH while the concentration of Dissolved Oxygen (DO) was regulated by varying the speed of the stirring impellers and by the flow of filtered air. When the fermentation finished, the cells were harvested by centrifugation and then snap-frozen in liquid nitrogen to be stored at -80°C. For the purification, the cells were lysed using 50 mM Tris-HCl pH 8.5 plus additives [3]. Before the first chromatography step, the buffer of the supernatant was exchanged to 10 mM sodium phosphate (NaPi) pH 6.0 using dialysis for volumes below 500 mL or tangential flow filtration (TFF) for larger volumes. This solution was then loaded to a bioprocess media (CaptoS, Cytiva). Hemoglobin was eluted in one step with 70 mM NaPi pH 7.2. As a second purification step, the anion exchange media QHP (GE Healthcare) was used. Before loading, the buffer was exchanged to 20mM Tris-HCl pH 8.3. Hemoglobin was eluted with a 10-column volume gradient of 50 mM sodium phosphate, pH 7.2, 100 mM sodium chloride.

For the animal studies, endotoxins were removed from the purified samples using a Proteus NoEndo™ Spin Column Kit (ProteinArk) according to the instructions given in the manual. The levels of endotoxin were determined by Lonza Biotech (Belgium) using the LAL Kinetic chromogenic assay. Those samples with levels of endotoxin below 5.0 EU/mL were converted to oxyhemoglobin (as described in the methods) and PEGylated by the Euro-PEG-Hb protocol under deoxy conditions as described previously [4]. This method yielded an average of 8.9 PEG molecules per tetramer for the F45 mutant.

References

1. Simons, M. et al. Comparison of the oxidative reactivity of recombinant fetal and adult human hemoglobin: implications for the design of hemoglobin-based oxygen carriers. *Biosci Rep.* **38**(2018).

2. Looker, D., Mathews, A.J., Neway, J.O. & Stetler, G.L. Expression of recombinant human hemoglobin in Escherichia coli. *Methods Enzymol* **231**, 364-74 (1994).
3. Leiva Eriksson, N., Reeder, B.J., Wilson, M.T. & Bulow, L. Sugar beet hemoglobins: reactions with nitric oxide and nitrite reveal differential roles for nitrogen metabolism. *Biochem J* **476**, 2111-2125 (2019).
4. Portoro, I. et al. Towards a novel haemoglobin-based oxygen carrier: Euro-PEG-Hb, physico-chemical properties, vasoactivity and renal filtration. *Biochim. Biophys. Acta* **1784**, 1402-9 (2008).
