# Supplementary material for: Taming hemoglobin chemistry—a new hemoglobin-based oxygen carrier engineered with both decreased rates of nitric oxide scavenging and lipid oxidation
Source: Exp Mol Med. 2024 Oct 1;56(10):2260–70. doi: 10.1038/s12276-024-01323-x (PMC11542024; doi:10.1038/s12276-024-01323-x)
Supplement: Supplementary file 3 — Supplementary Figures (animal studies) [file 12276_2024_1323_MOESM3_ESM.pdf]

SUPPLEMENTARY FIGURES (ANIMAL STUDIES)

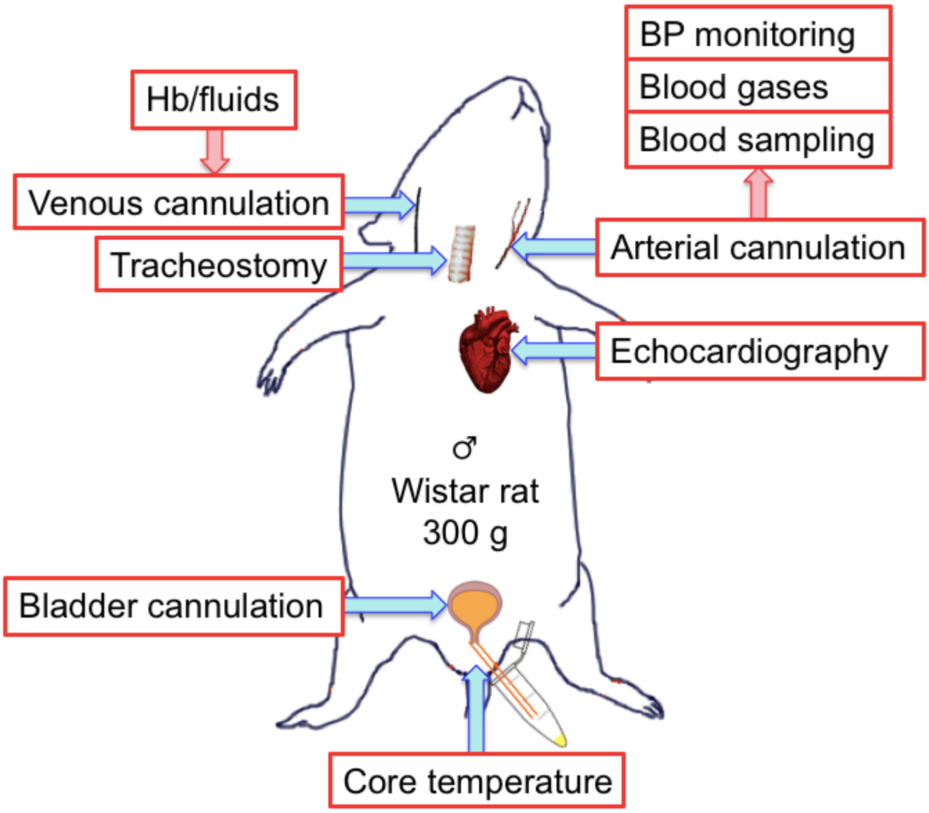

Supplementary Fig. 1
Illustration of surgical instrumentation with cardiorespiratory monitoring
BP, blood pressure

Study 1 and 2 Hb, hemoglobin; PK, pharmacokinetics; V, vehicle (Ringer's lactate)

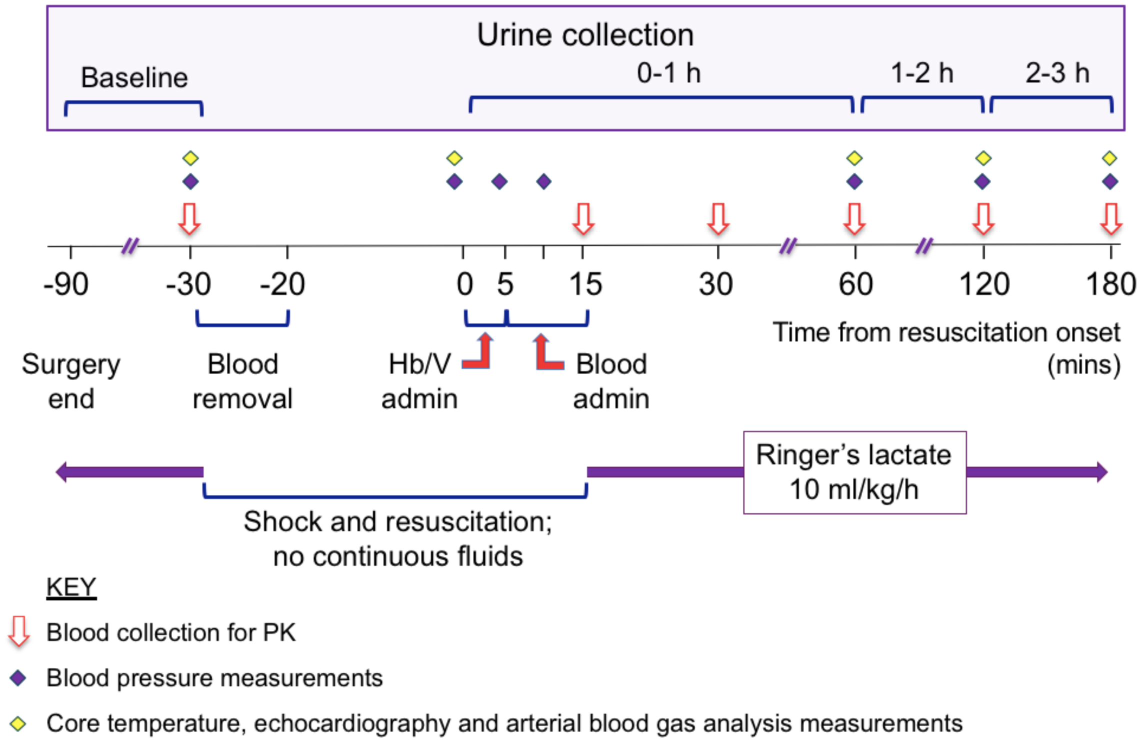

Study 3 Hb, hemoglobin; RL, Ringer's lactate; V, vehicle (Ringer's lactate)

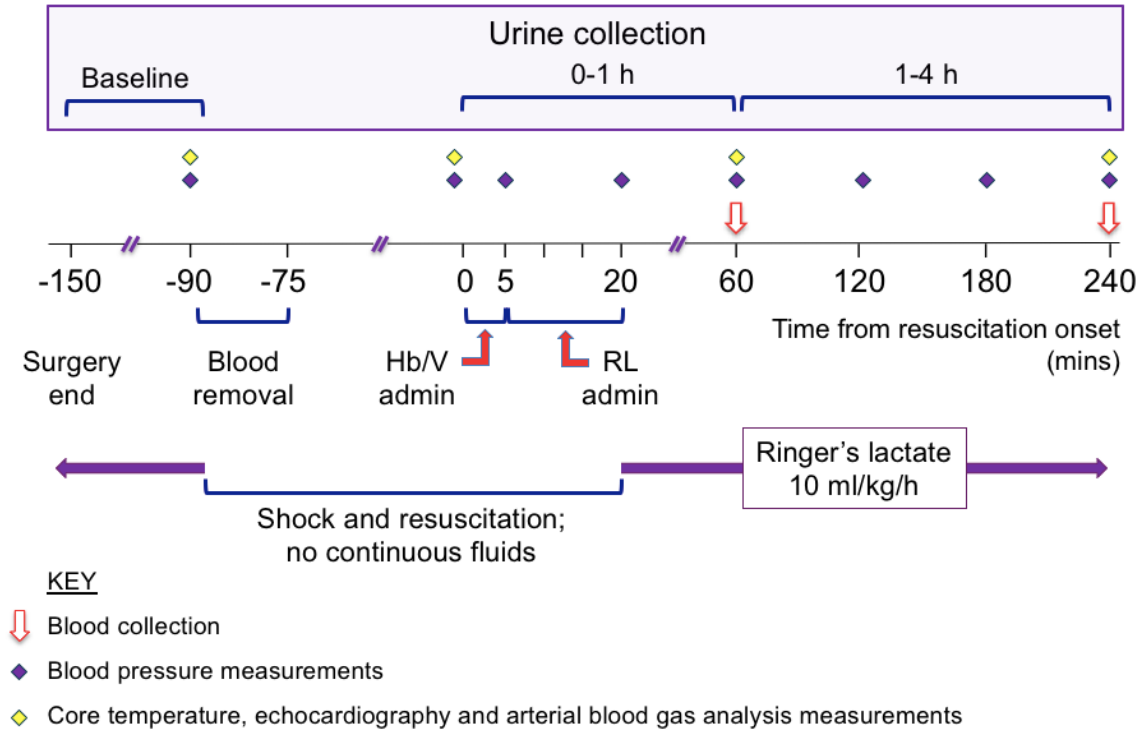

Supplementary Fig. 2 Experimental protocols for animal studies

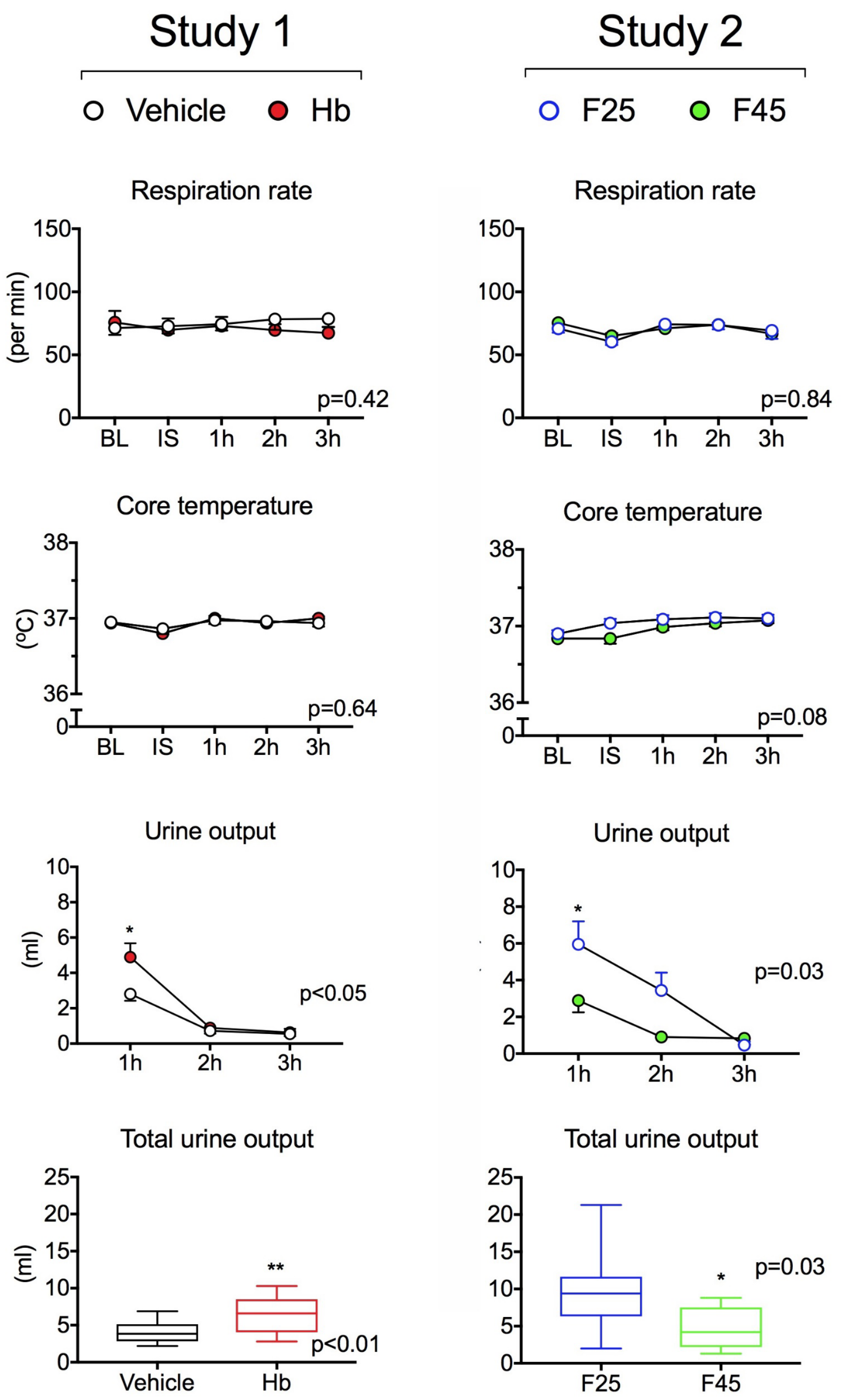

Supplementary Fig. 3 Basic Physiology

BL, baseline; IS, ischemia; Hb, hemoglobin. All times stated are from the onset of resuscitation. *p<0.05, **p<0.01.

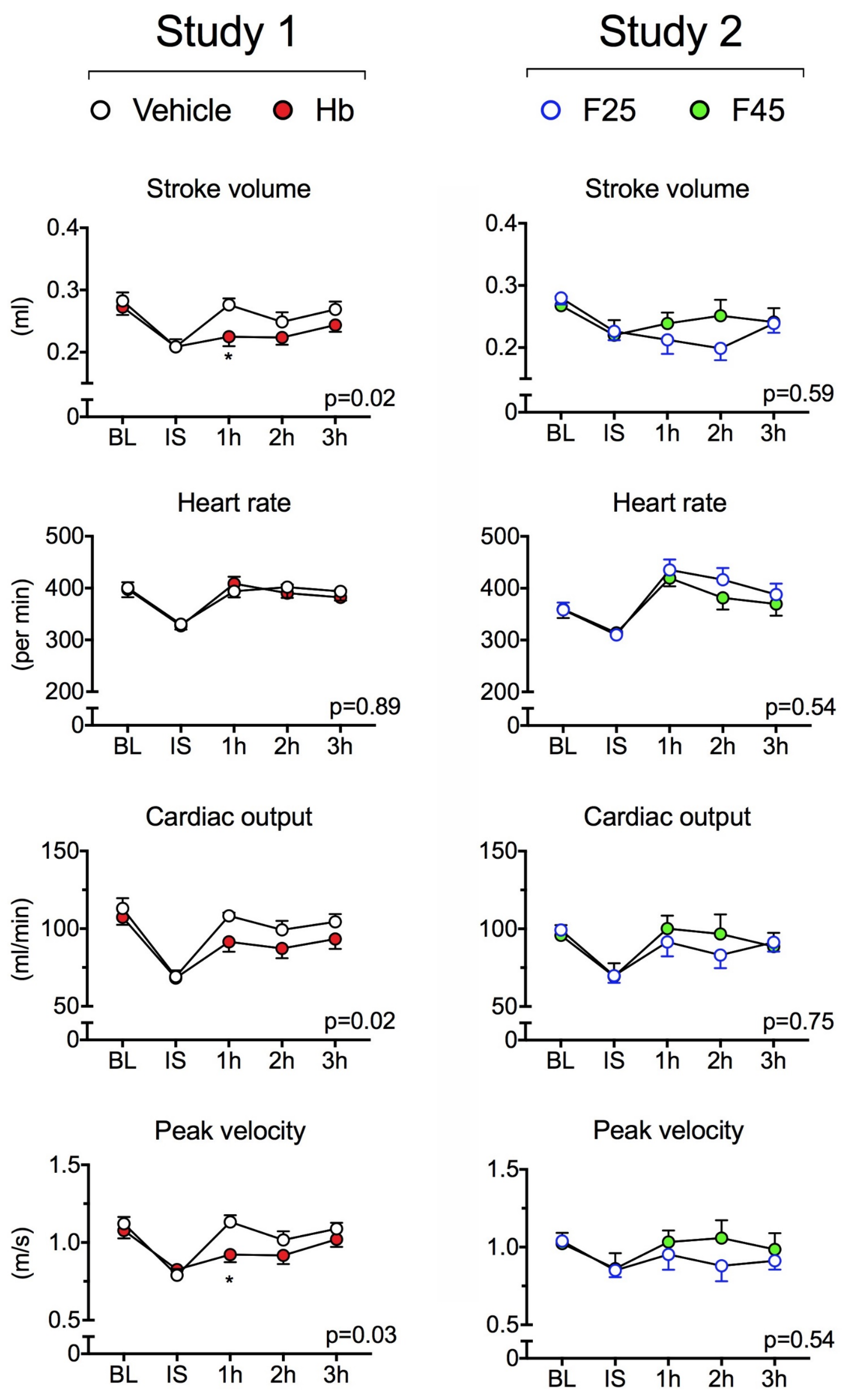

Supplementary Fig. 4 Cardiac function.
BL, baseline; IS, ischaemia; Hb, hemoglobin. All times stated are from the onset of resuscitation. *p<0.05.

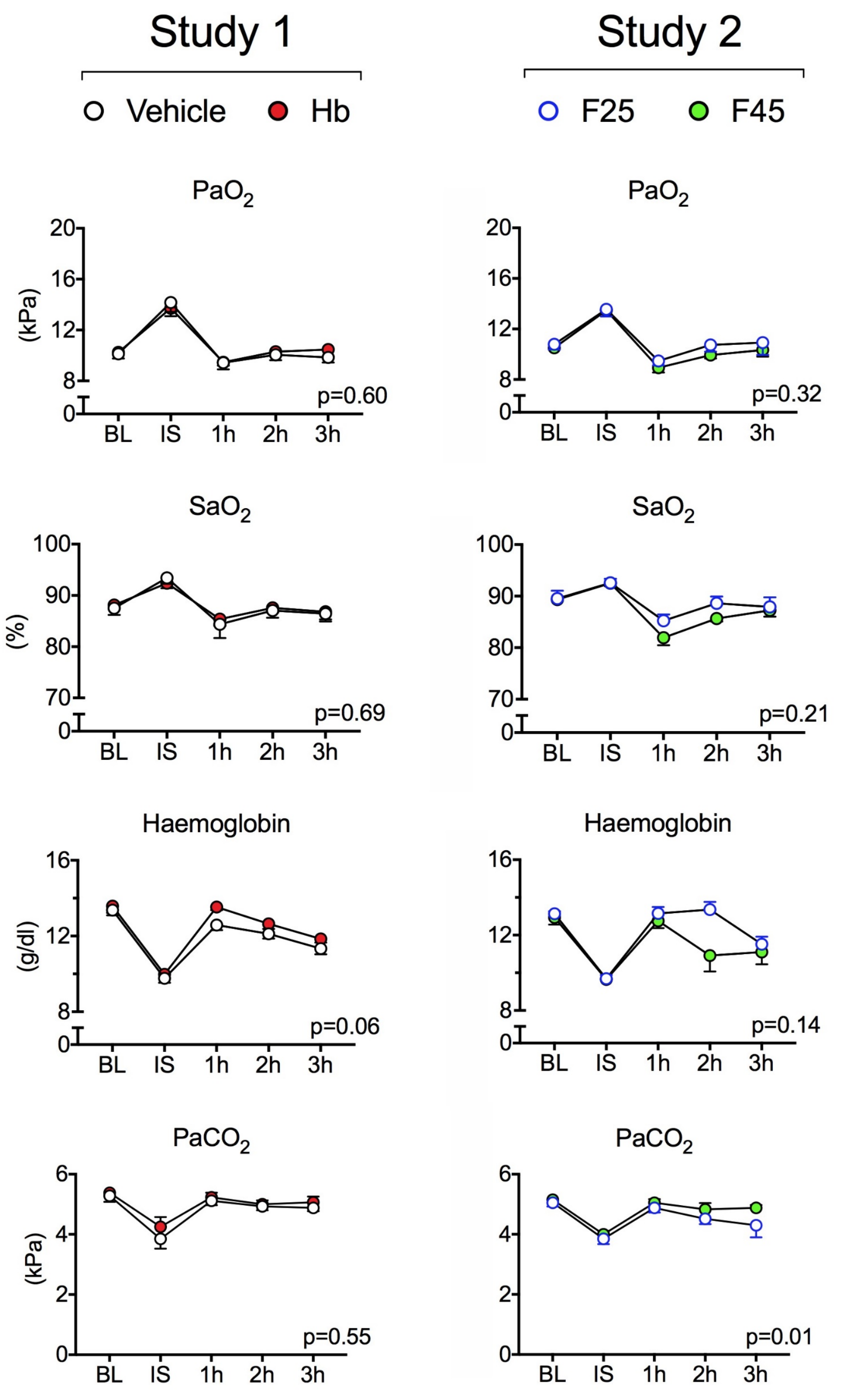

Supplementary Fig. 5 Arterial blood gas analysis.
BL, baseline; IS, ischemia; Hb, hemoglobin; PaO_2 and $PaCO_2$ are the arterial partial pressures of, respectively, oxygen and carbon dioxide; SaO_2, hemoglobin oxygen saturation. All times stated are from the onset of resuscitation.

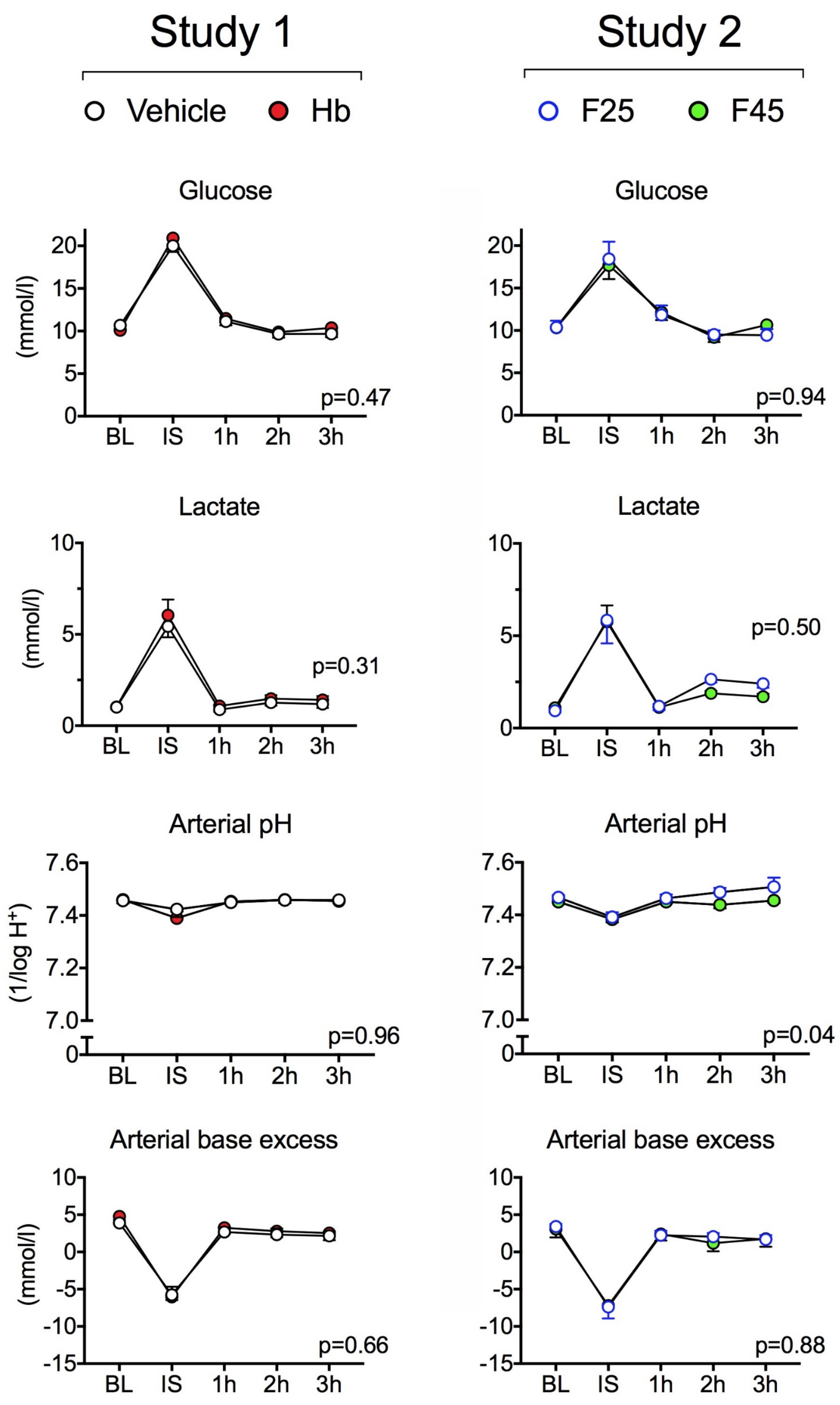

Supplementary Fig. 6 Glycaemic status, organ perfusion and acid-base status. BL, baseline; IS, ischemia; Hb, hemoglobin. All times stated are from the onset of resuscitation.

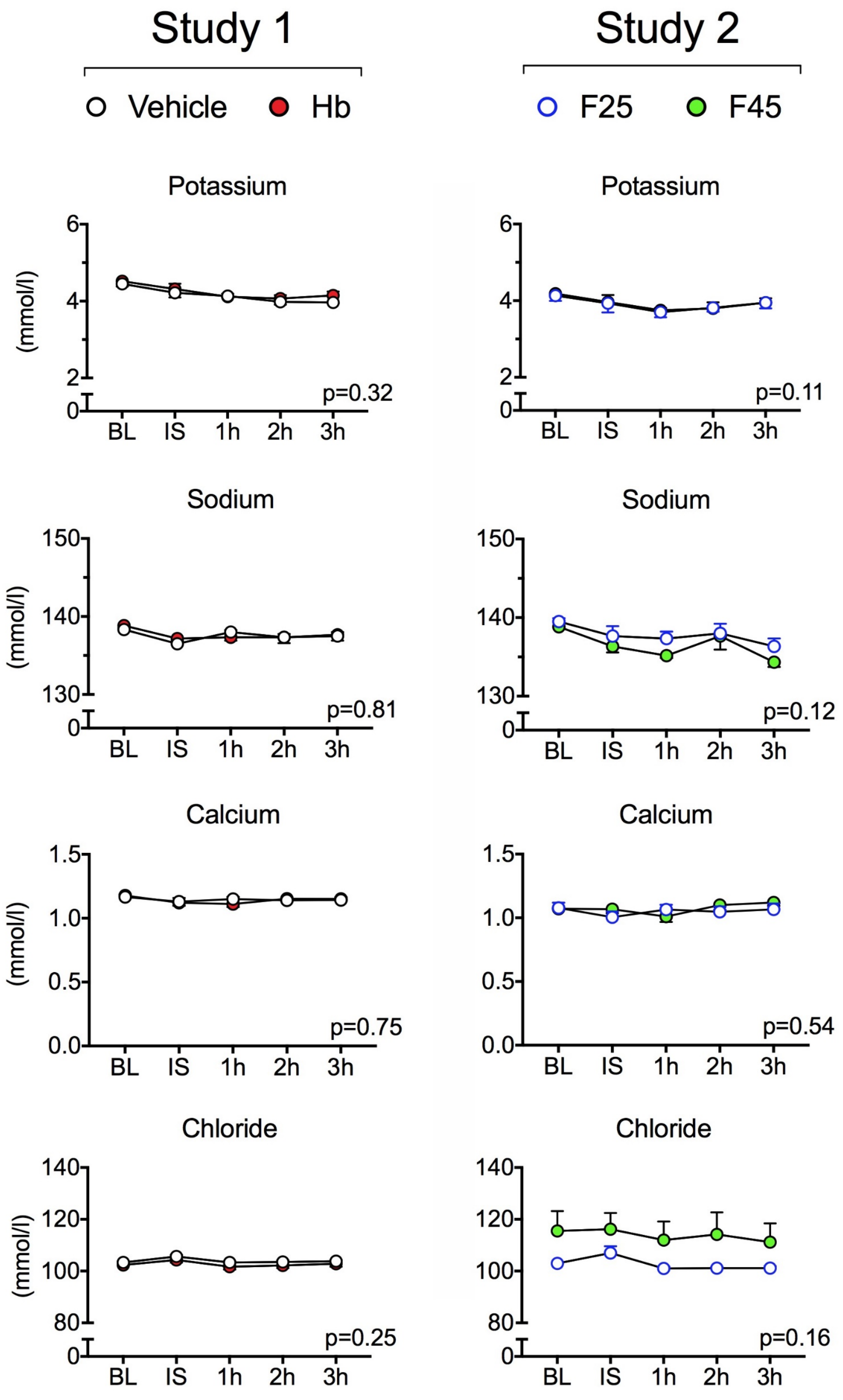

Supplementary Fig. 7 Electrolytes.
Electrolytes. BL, baseline; IS, ischemia; Hb, hemoglobin. All times stated are from the onset of resuscitation.

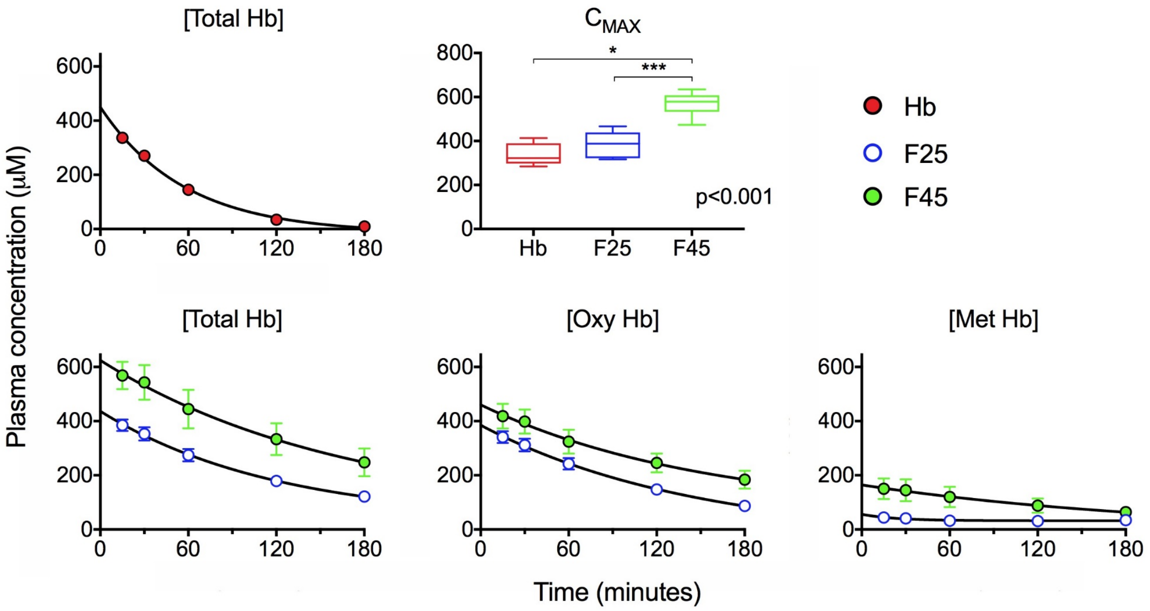

Supplementary Fig. 8 Pharmacokinetic distribution.
This could be accurately modelled using a one-phase decay curve indicative of a one-compartment model. C_{MAX}, maximum plasma concentration; Hb, hemoglobin; Met, methemoglobin; Oxy, oxyhemoglobin. Note that 1) these data refer solely to Hb-treated animals and 2) there was no Met Hb in (human) Hb-treated animals meaning [Total Hb] = [Oxy Hb] in study 1 (top left). The C_{MAX} relates to [Total Hb] in both studies. All times stated are from the onset of resuscitation. All times stated are from the onset of resuscitation. *p<0.05, **p<0.01. ***p<0.001.

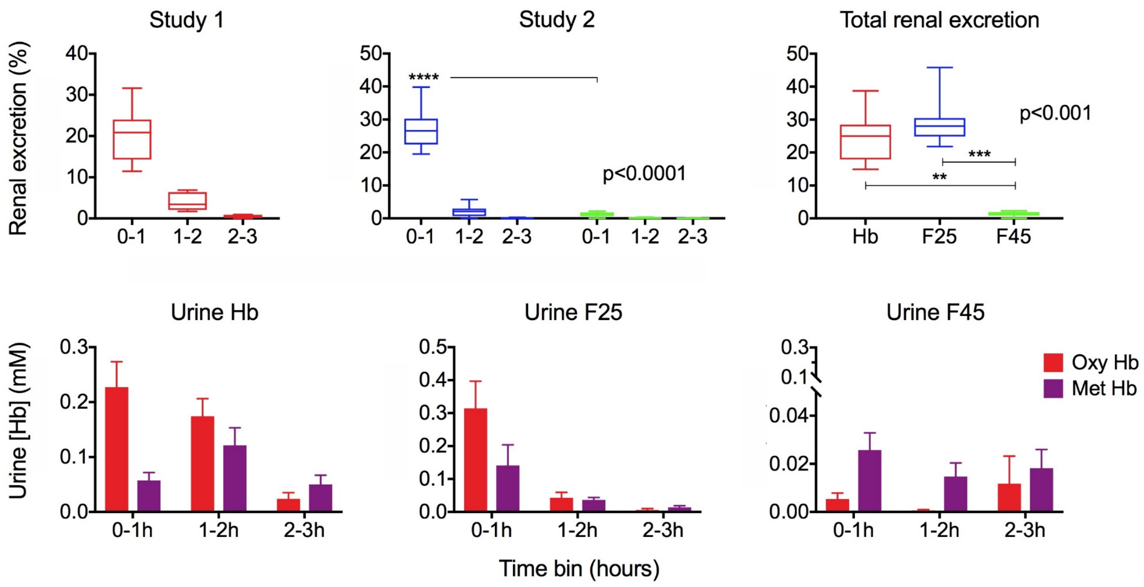

Supplementary Fig. 9 Renal excretion.
Hb, hemoglobin. **p<0.01, ***p<0.001, ****p<0.0001

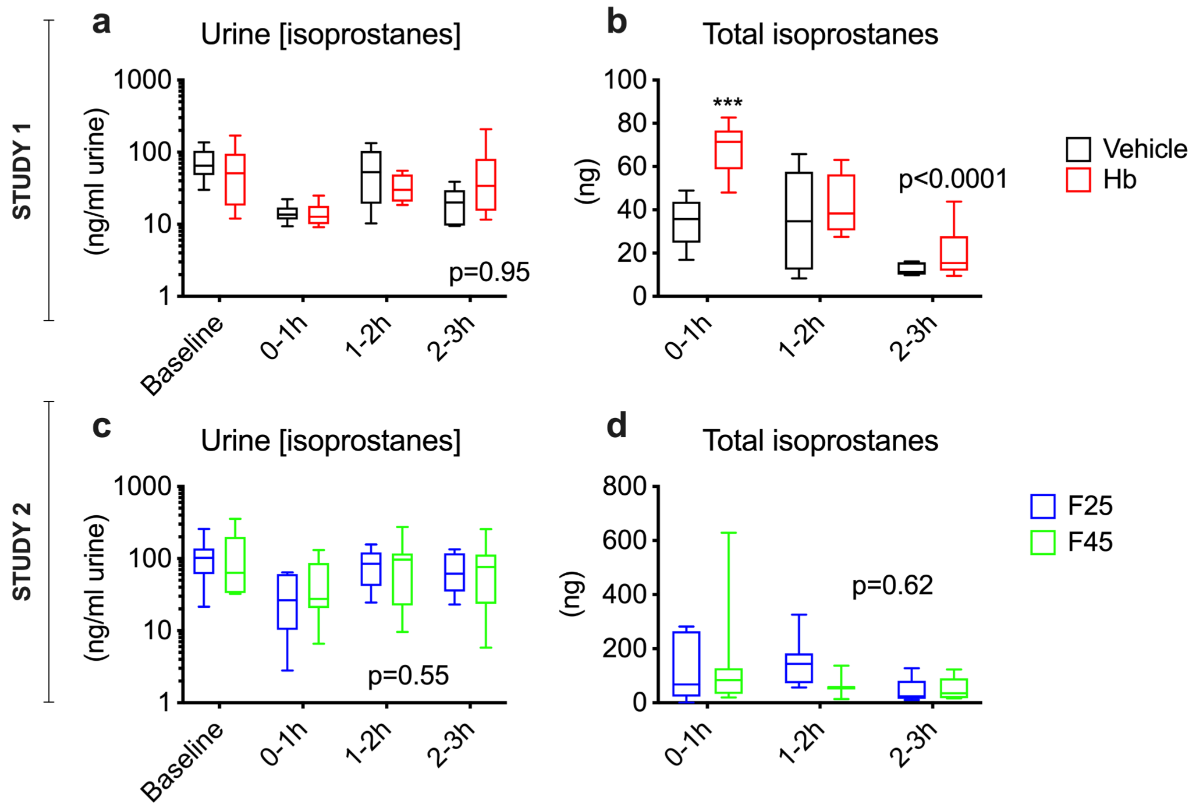

Supplementary Fig. 10 Urinary isoprostanes.
Time-dependent concentrations (left panels); corrected for variations in urine output to give total urinary isoprostanes (right panel). ***p<0.0001
